# Intervalence Charge Transfer in Nonbonding, Mixed-Valence, Homobimetallic Ytterbium Complexes

**DOI:** 10.1021/jacs.3c13906

**Published:** 2024-02-19

**Authors:** Michael D. Roy, Thaige P. Gompa, Samuel M. Greer, Ningxin Jiang, Lila S. Nassar, Alexander Steiner, John Bacsa, Benjamin W. Stein, Henry S. La Pierre

**Affiliations:** † School of Chemistry and Biochemistry, Georgia Institute of Technology, Atlanta, Georgia 30332-0400, United States; ‡ Los Alamos National Laboratory, Los Alamos, New Mexico 87545, United States; § School of Physics, Georgia Institute of Technology, Atlanta, Georgia 30332-0400, United States; ∥ Department of Chemistry, University of Liverpool, Liverpool L69 7Zd, United Kingdom; ⊥ Nuclear and Radiological Engineering Program, Georgia Institute of Technology, Atlanta, Georgia 30332-0400, United States; # Physical Sciences Division, Pacific Northwest National Laboratory, Richland, Washington 99352, United States

## Abstract

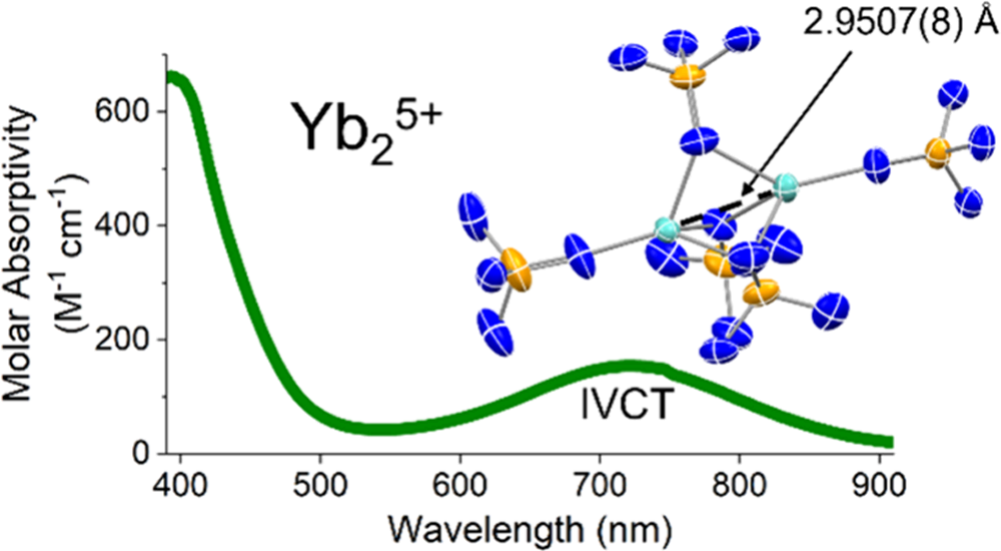

There are several reports of compounds containing lanthanide ions in two different formal oxidation states; however, there are strikingly few examples of intervalence charge transfer (IVCT) transitions observed for these complexes, with those few occurrences limited to extended solids rather than molecular species. Herein, we report the synthesis, characterization, and computational analysis for a series of ytterbium complexes including a mixed-valence Yb_2_^5+^ complex featuring a remarkably short Yb···Yb distance of 2.9507(8) Å. In contrast to recent reports of short Ln···Ln distances attributed to bonding through 5*d* orbitals, the formally Yb_2_^5+^ complex presented here displays clear localization of Ln^2+^ and Ln^3+^ character and yet still displays an IVCT in the visible spectrum. These results demonstrate the ability to tune the electronic structure of formally mixed oxidation state lanthanide complexes: the high exchange stabilization of the Yb^2+^ 4*f*^14^ configuration disfavors the formation of a 5*d*^1^ bonding configuration, and the short metal–metal distance enforced by the ligand framework allows for the first observed lanthanide IVCT in a molecular system.

## Introduction

Mixed-valence compounds with intervalence charge transfer (IVCT) transitions are known across the periodic table, although to date, IVCTs between lanthanide ions have only been reported in extended solid materials.^1−3^ Given the propensity for lanthanide ions to favor the +3 oxidation state, it is unsurprising that there are few examples of mixed-valence molecular complexes of lanthanides.^4−11^ While some of these complexes have large separations between the Ln^2+^ and Ln^3+^ ions, others have closer contact (below ∼3.5 Å), yet IVCT transitions are not observed. These complexes feature reduced Sm^2+^, Eu^2+^, or Yb^2+^, which have purely 4*f* valence electron configurations.^12^ In contrast, reduction of lanthanides to mixed 4*f*/5*d* configurations results in dramatically different properties, such as metal–metal bonding and high magnetic coercivity.^13^ Related lanthanide complexes have been reported in recent years,^14−16^ as well as a related metal–metal bonded trithorium cluster.^17^ The abundance of mixed-valence complexes with IVCT transitions outside of the *f*-block, and the depth of study these compounds have received, contrasts with few examples of mixed-valence lanthanide compounds.^18−22^ Given the recent reports of mixed-valence and metal–metal bonded lanthanide^13−16^ and actinide^17^ compounds, it is important to build a cohesive understanding of the existing literature and these novel materials.

The possibility of lanthanide–lanthanide bonding complicates the assignment of IVCT features. Electronic transitions may result either from genuine charge transfer between ions of different formal oxidation state, as in the Robin-Day Class II categorization,^19^ or from bonding-to-antibonding transitions, as is well described for *d*-block metal–metal bonded compounds^23^ and apparent in the recently reported Ln–Ln bonded systems.^13^ Importantly, the Robin-Day classification scheme does not consider direct metal–metal bonding. However, it is common practice in the literature for metal–metal bonded systems to be described as Robin-Day Class III systems and the bonding-to-antibonding transitions to be described as IVCT despite the ions sharing equal, intermediate valence with no charge transfer upon excitation. This conflation of definitions can lead to ambiguous or inaccurate assignments of these spectroscopic features. With this understanding, one can consider three scenarios for a formally mixed-valence Ln_2_^5+^ system:

 

 

1. Ln–Ln bonding with one delocalized 5*d* electron, a σ → σ* transition, and intermediate valence (i.e., Ln^2.5+^).
2. Charge-localized noninteracting ions, as in the Robin-Day Class I categorization, which do not display any IVCT.
3. Charge-localized ions with 4*f* → 4*f* IVCT, as in the Robin-Day Class II or Class II/III categorization.

Scenario 2 is easily identified by the absence of an electronic transition other than the typical *f* → *f* and *f* → *d* transitions associated with the individual Ln^2+^ and Ln^3+^ ions. Scenarios 1 and 3 cannot be readily distinguished by their UV/vis/NIR spectra; however, the occupation of a delocalized bonding orbital has a dramatic influence on the ground state magnetic behavior compared to the charge localized systems.^13,24,25^ Therefore, a combination of electronic absorption spectroscopy and magnetometry permits for the unique assignment of an electronic structure for mixed-valence lanthanide complexes.

Herein, we report the synthesis and characterization of some molecular homobimetallic compounds containing ytterbium in both trivalent and divalent oxidation states. The mixed-valence compound [Yb_2_(NP(pip)_3_)_5_] (**3-[Yb**_**2**_**]**^**5+**^, NP(pip)_3_ = tri(piperidinyl) imidophosphorane) features a remarkably short Yb···Yb distance of 2.9507(8) Å. Through analysis of single-crystal X-ray structural data, electronic absorption spectroscopy, and dc SQUID magnetometry combined with theoretical analysis, it is evident that there is no metal–metal bond despite the short internuclear distance. The presence of an IVCT and a clearly mixed-valence Yb^2+^/Yb^3+^ ground state lead to the classification of this complex as a Robin-Day Class II complex. Importantly, this system demonstrates that short internuclear distance, combined with a purely 4*f* valence configuration, enables lanthanide-based IVCT. Conversely, this result sets a new limit on short-distance nonbonding interactions between lanthanide ions. When paired with recent results on lanthanide–lanthanide bonding, this system highlights both the role of *d* orbital occupation in forming metal–metal bonds in molecular *f*-block complexes^13,15−17^ and the origins of spectroscopic features traditionally described as IVCT transitions.

## Results

### Synthesis and Molecular Structures

**Scheme 1:**
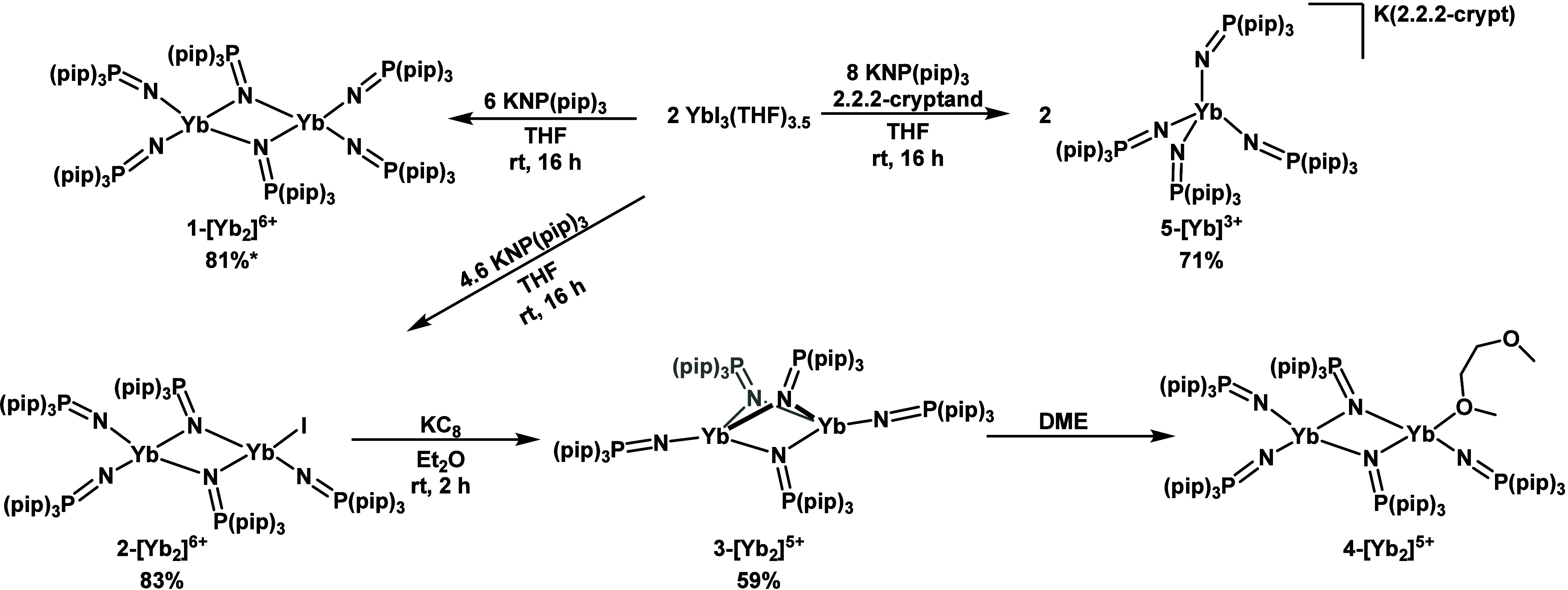
Synthesis of Compounds **1**–**5**

The compounds reported in this work are supported by the previously reported tri(piperidinyl)imidophosphorane ligand, [NP(pip)_3_]^1–^.^26^ The syntheses of each of these compounds are depicted in Scheme 1. The dimeric Yb^3+^ complex [Yb_2_(NP(pip)_3_)_6_], **1-[Yb**_**2**_**]**^**6+**^, and the monomeric Yb^3+^ complex [K([2.2.2]cryptand)][Yb(NP(pip)_3_)_4_], **5-[Yb]**^**3+**^, are synthesized through salt metathesis of YbI_3_(THF)_3.5_ (THF = tetrahydrofuran) with 3 or 4 equiv of the ligand salt K[NP(pip)_3_], respectively. Addition of stoichiometric [2.2.2]crypt and following the initial salt metathesis results in the outer-sphere potassium ion in the charge-separated salt **5-[Yb]**^**3+**^. Synthesis of [Yb_2_(NP(pip)_3_)_5_I], **2-[Yb**_**2**_**]**^**6+**^, requires careful control of the addition conditions to prevent formation of **1-[Yb**_**2**_**]**^**6+**^ along with unreacted YbI_3_(THF)_3.5_. A stepwise addition is used, where approximately 1 equiv of dissolved ligand is added directly to a vigorously stirring slurry of YbI_3_(THF)_3.5_ in THF, with the remaining ∼1.5 equiv added dropwise over 10 min. The mixed-valence complex [Yb_2_(NP(pip)_3_)_5_], **3-[Yb**_**2**_**]**^**5+**^, results from the reduction of **2-[Yb**_**2**_**]**^**6+**^ with KC_8_. Recrystallization of **3-[Yb**_**2**_**]**^**5+**^ from the coordinating solvent 1,2-dimethoxyethane (DME) results in isolation of the solvent adduct [Yb_2_(DME)(NP(pip)_3_)_5_], **4-[Yb**_**2**_**]**^**5+**^.

Further reduction to a [Yb_2_]^4+^ complex was explored. Reaction of **2-[Yb**_**2**_**]**^**6+**^ with excess KC_8_ results in **3-[Yb**_**2**_**]**^**5+**^ as the only isolated product; therefore, electrochemical reduction was also explored. Previous efforts toward electrochemical analysis on cerium complexes supported by the [NP(pip)_3_]^1–^ ligand resulted in complete degradation of the complexes,^26^ and similar decomposition was initially observed for the complexes presented here in THF with [^*n*^Bu_4_N][PF_6_] electrolyte. Recently reported methods, notably the isolation of the silver electrode within a fritted capillary and the use of tetrabutylammonium tetraphenylborate as the supporting electrolyte in THF, have enabled electrochemical studies on other *f*-element complexes supported by imidophosphorane ligands.^27,28^ When these methods are employed, a reduction of **3-[Yb**_**2**_**]**^**5+**^ is observed at −2.3 V vs Fc^+/0^. This reduction is chemically quasi-reversible and electrochemically irreversible and shows evidence of chemical decomposition on the time scale of the cyclic voltammetry measurements (see Figure S1, Table S1), consistent with the chemical inaccessibility of a [Yb_2_]^4+^ species.

**Figure 1:**
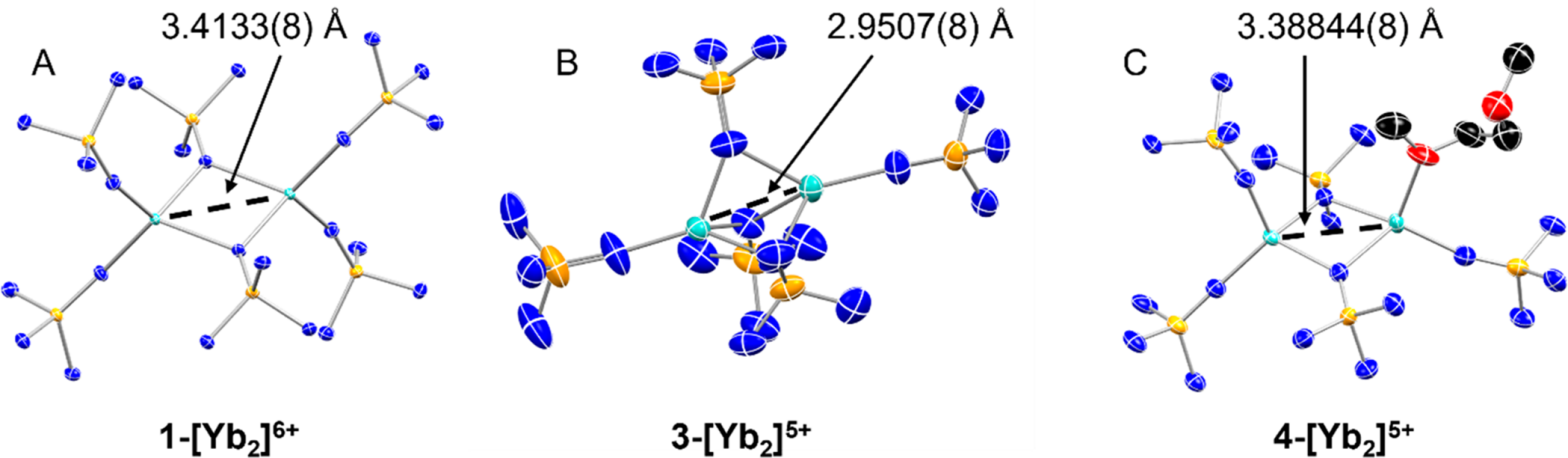
Single-crystal X-ray diffraction determined molecular structures of (A) **1-[Yb**_**2**_**]**^**6+**^, (B) **3-[Yb**_**2**_**]**^**5+**^, and (C) **4-[Yb**_**2**_**]**^**5+**^. Thermal ellipsoids are shown at 50% probability with cocrystallized solvent, hydrogen, and piperidinyl carbon atoms omitted for clarity.

The solid-state structural features of these ytterbium complexes were determined by single-crystal X-ray diffraction. The space group and selected bonding distances for each complex are summarized in Table 1. Structural representations of **1-[Yb**_**2**_**]**^**6+**^, **3-[Yb**_**2**_**]**^**5+**^, and **4-[Yb**_**2**_**]**^**5+**^ are given in Figure 1. Both **1-[Yb**_**2**_**]**^**6+**^ and **2-[Yb**_**2**_**]**^**6+**^ feature Yb_2_N_2_ diamond cores with a combination of bridging and terminal imidophosphorane ligands. Due to the geometric constraints of the bridging ligands, the Yb···Yb distance is 3.4133(6) Å in **1-[Yb**_**2**_**]**^**6+**^ and 3.3679(6) Å in **2-[Yb**_**2**_**]**^**6+**^. These distances are shorter than recently reported 1/2-order bonds in earlier lanthanides.^13^ When the reduced Yb_2_^5+^ complex is crystallized as the DME adduct, **4-[Yb**_**2**_**]**^**5+**^, it adopts a similar geometry with an Yb···Yb separation of 3.38844(8) Å. In this case, metal–ligand bond distances at each of the metal centers suggest charge localization as distinct Yb^2+^ and Yb^3+^ centers.

**Table 1:** Selected Bond Distances for **1**–**5**

Compound	Space Group	Yb···Yb (Å)	FSR′	Yb–N_term_ (Å)	Yb–N_brid_ (Å)	Yb–X (Å)
**1-[Yb**_**2**_**]**^**6+**^	*P*2_1_/*n*	3.4133(8)^a^	0.983	2.1342[2]^a^	2.2739[2]	N/A
**2-[Yb**_**2**_**]**^**6+**^	*P*1	3.3679(6)	0.970	2.130[2] (homoleptic)	2.282[3] (homoleptic)	2.9777(5)
				2.0648(12) (heteroleptic)	2.2179[2] (heteroleptic)	
**3-[Yb**_**2**_**]**^**5+**^	*P*2_1_	2.9507(8)	0.850	2.118(15) (Yb^3+^)	2.27[2] (Yb^3+^)	N/A
				2.183(12) (Yb^2+^)	2.39[2] (Yb^2+^)	
**4-[Yb**_**2**_**]**^**5+**^	*P*2_1_2_1_2_1_	3.38844(8)	0.976	2.146[9] (Yb^3+^)	2.272[8] (Yb^3+^)	2.358(9)
				2.216(6) (Yb^2+^)	2.378[9] (Yb^2+^)	
**5-[Yb]**^**3+**^	*P*1	N/A	N/A	2.177[3]	N/A	N/A

a ESDs for individual values are in (parentheses) while ESDs for averages, calculated by addition in quadrature, are in [square brackets].

The homoleptic mixed-valence complex **3-[Yb**_**2**_**]**^**5+**^ exhibits a different structural motif with three bridging imidophosphorane ligands and one terminal ligand per ytterbium ion. The Yb···Yb distance in **3-[Yb**_**2**_**]**^**5+**^ is even shorter than that in the other Yb_2_ complexes at only 2.9507(8) Å. This distance is 0.4626 and 0.4172 Å shorter than the distances in **1-[Yb**_**2**_**]**^**6+**^ and **2-[Yb**_**2**_**]**^**6+**^ despite the expected increase of 0.15 Å in the ionic radius upon reduction from trivalent to divalent ytterbium. While this distance is still longer than the sum of the Shannon ionic radii (1.888 Å),^29^ it is well below the Pyykkö covalent distance of 3.40 Å.^30^ This Yb···Yb distance, the shortest of any Yb···Yb distance reported in the Cambridge Structural Database (CSD)^31^ and among the shortest Ln···Ln distances reported,^32^ suggests the possibility of a strong metal–metal interaction or even a metal–metal bond. However, the two ions are not crystallographically equivalent: distinct Yb–N bond lengths are observed with the average Yb–N distance to bridging ligands differing by 0.12 Å between the two metal centers, indicating charge localization as Yb^2+^ and Yb^3+^. Thus, while the Yb···Yb distance suggests the possibility of a strong Yb–Yb interaction, the charge separation indicates a Robin-Day Class I, II, or II/III mixed-valence configuration.

To better contextualize the relative lengths of the internuclear distances across the lanthanide series, the distances must be normalized to the elements involved. This methodology was the concept behind the formal shortness ratio, FSR, defined by Cotton;^33,34^ however, we find the radii selected by Cotton to be a poor fit for lanthanides. We therefore propose a modified FSR′ based on the Shannon ionic radii.^29^ (See Supporting Information (SI) for an extended discussion on metal–metal distances.) Compounds **3-[Yb**_**2**_**]**^**5+**^ and **4-[Yb**_**2**_**]**^**5+**^ have FSR′ values of 0.850 and 0.976, respectively. In comparison, the two-center one-electron bonded systems reported for gadolinium, terbium, and dysprosium have FSR′ values of 1.00, 1.01, and 1.02, respectively.^13^ A review of short Ln···Ln distances reported in the CSD reveals several with FSR′ values well below 1.00, including the 2.9270(6) Å distance in [(Me_4_TACD)_2_Lu_2_(μ-H)_4_][BAr^F^-24]_2_ (FSR′ = 0.850, Me_4_TACD = 1,4,7,10-tetramethyl-1,4,7,10-tetraazacyclododecane).^32^ Short FSR′ values are also seen for complexes with divalent or tetravalent lanthanide ions, such as the Sm^2+^ bimetallic complex [(OEPG)Sm_2_(Et_2_O)_2_] (FSR′ = 0.865, OEP = octaethylporphyrinogen)^35^ and the Ce^4+^ dimer K_4_[Ce(O_2_)(EDTA)]_2_·14H_2_O (FSR′ = 0.791, EDTA = ethylenediaminetetreaacetate).^36^ These examples, along with several others provided in the Supporting Information, illustrate both the difficulty in comparing non-normalized internuclear distances and the poor correlation between internuclear distance and metal–metal bonding.

### Electronic Absorption Spectroscopy

**Figure 2:**
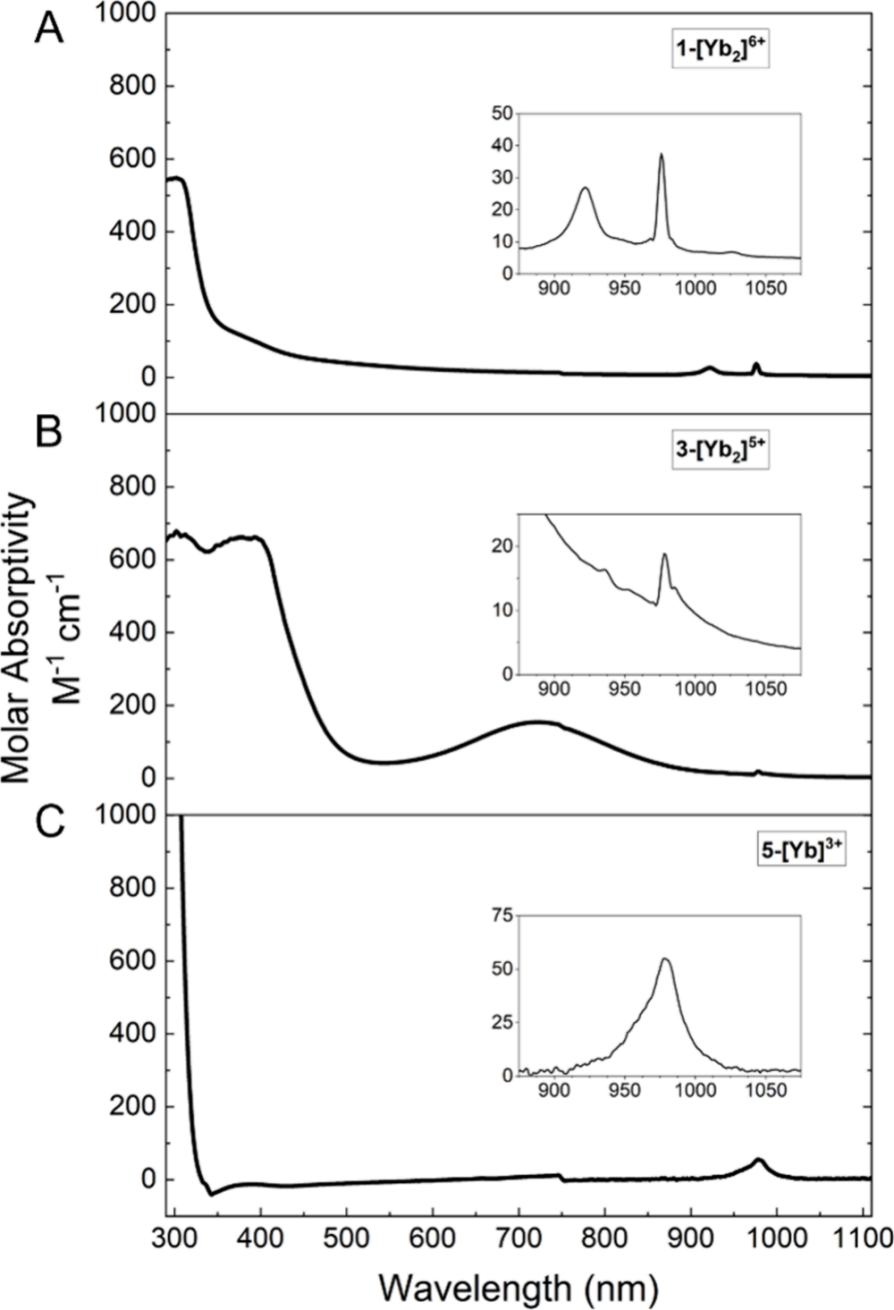
UV/vis/NIR electronic absorption spectra for **1-[Yb**_**2**_**]**^**6+**^ (A), **3-[Yb**_**2**_**]**^**5+**^ (B), and **5-[Yb]**^**3+**^ (C) in toluene at ambient temperature. Insets show the *f* → *f* transitions in the NIR region.

Compounds **1-[Yb**_**2**_**]**^**6+**^ and **5-[Yb]**^**3+**^ are colorless, and compound **2-[Yb**_**2**_**]**^**6+**^ is pale yellow. There are no observable transitions in the visible range of the electronic absorption spectra of these compounds, as seen in the UV/vis/NIR spectra for **1-[Yb**_**2**_**]**^**6+**^ and **5-[Yb]**^**3+**^ in Figure 2. The shoulder of a charge transfer feature is observed at high energy, with very broad, weak absorbance in the visible spectrum, accounting for the white/yellow color of these compounds. All three additionally feature one or two low intensity, sharp *f* → *f* transitions in the near-IR, between 900 and 1000 nm. In **5-[Yb]**^**3+**^, only a single, relatively broad transition is observed for this ^2^F_7/2_ → ^2^F_5/2_ transition, as the crystal field splitting is washed out by the line width of the transition. Narrower line widths, attributed to increased rigidity of the bimetallic complexes, allow for resolution of two transitions in **1-[Yb**_**2**_**]**^**6+**^ and **3-[Yb**_**2**_**]**^**5+**^. These transitions, separated by ∼600 cm^–1^ in **1-[Yb**_**2**_**]**^**6+**^ and ∼500 cm^–1^ in **3-[Yb**_**2**_**]**^**5+**^, arise from crystal field splitting of the ^2^F_7/2_ ground state and the ^2^F_5/2_ excited state of the Yb^3+^ cation. Extensive assignments of crystal field splitting for Yb^3+^ have been carried out for low-temperature solid-state samples, and the magnitude of splitting is similar to the splitting observed for **1-[Yb**_**2**_**]**^**6+**^ and **3-[Yb**_**2**_**]**^**5+**^.^37−39^ The presence of two Yb^3+^ ions in **1-[Yb**_**2**_**]**^**6+**^ invites the possibility of a cooperative transition from the [^2^F_7/2_, ^2^F_7/2_] ground state to a [^2^F_5/2_, ^2^F_5/2_] excited state;^38,39^ however, no such transitions are observed in the 450–500 nm region. It is not possible to determine whether these transitions are entirely absent or are obscured by the tail of the charge transfer feature in the UV region.

**Figure 3:**
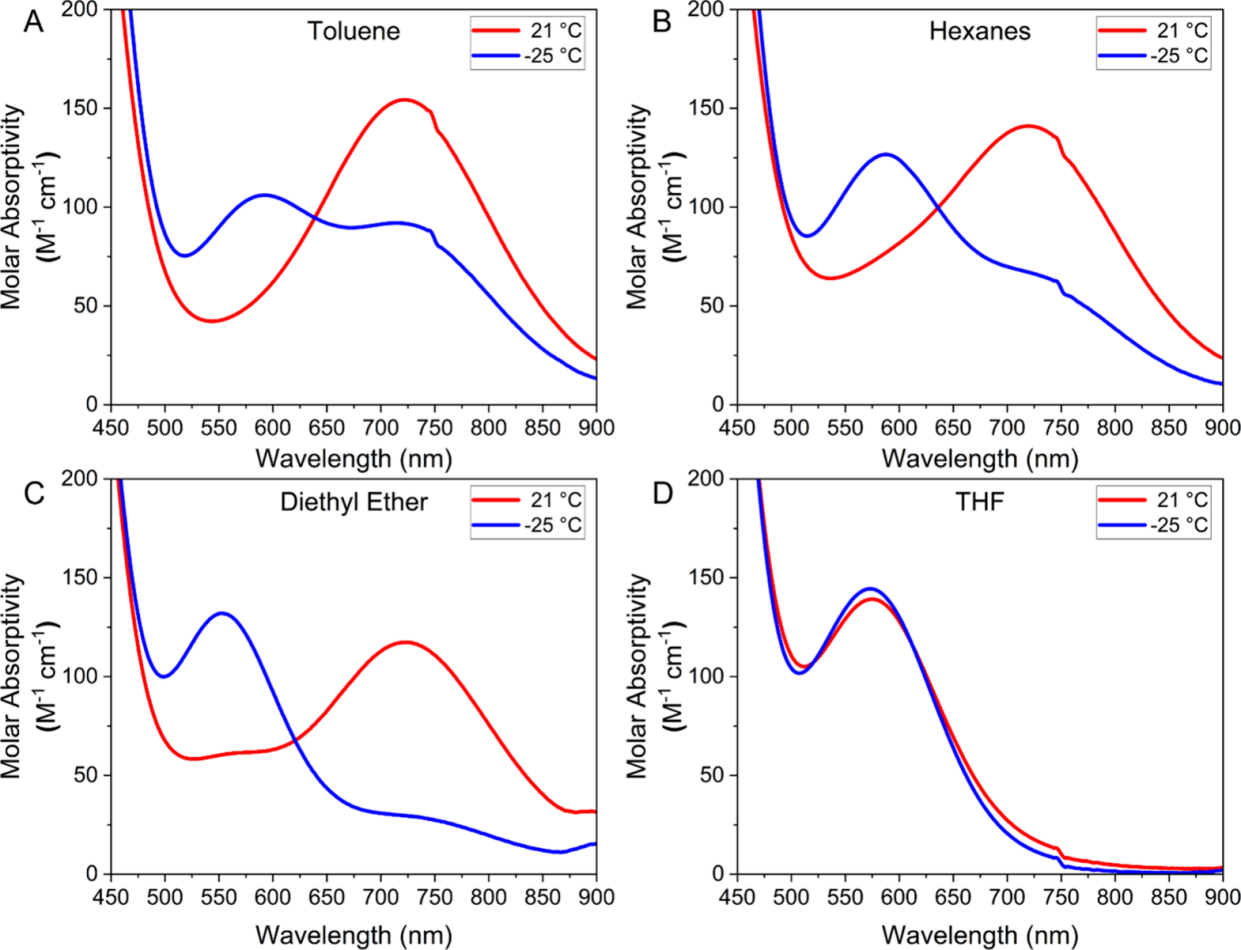
Temperature and solvent dependence of UV/vis/NIR electronic absorption spectra for **3-[Yb**_**2**_**]**^**5+**^ in toluene (A), hexanes (B), diethyl ether (C), and THF (D). Red traces are at 21 °C and blue traces are at −25 °C.

Compound **3-[Yb**_**2**_**]**^**5+**^ is intensely green and shows two additional spectral features. A higher energy, more intense peak (380 nm, 700 M^–1^ cm^–1^) can be attributed to an *f* → *d* transition (see also TDDFT analysis below), as is seen in other divalent ytterbium complexes.^40,41^ The lower energy, lower intensity feature (725 nm, 150 M^–1^ cm^–1^) is much broader, nearly obscuring the *f* → *f* transitions. Given the mixed-valence nature of **3-[Yb**_**2**_**]**^**5+**^, we can offer a preliminary assignment of an IVCT to this transition. To better understand this feature, spectra were acquired between −25 and +21 °C in hexanes, toluene, diethyl ether, and THF (Figure 3). The room temperature data in hexanes, toluene, and diethyl ether are comparable (red trace in Figure 3A–C). In contrast, the observed peak in THF (Figure 3D) is at 575 nm. Notably, the intensity of the ∼720 nm peak observed in hexanes, toluene, and diethyl ether at 21 °C decreases as the temperature is decreased, and a new feature emerges at ∼575 nm. This conversion is one-to-one as indicated by the clear isosbestic point in Figure S24. Based on the isolation of the solvated open form **4-[Yb**_**2**_**]**^**5+**^ from DME, we can infer that the ∼575 nm peak corresponds to this open form that is observed exclusively in THF, while the ∼720 nm peak corresponds to the closed form seen in the crystal structure of **3-[Yb**_**2**_**]**^**5+**^. Further, in noncoordinating or weakly coordinating solvents, these two isomers exist in a temperature-dependent equilibrium, with the closed form being favored at higher temperatures.

To facilitate analysis of the putative IVCT transitions, the two peaks were modeled as individual Gaussian peaks of the functional form (see SI for details of fitting in cm^–1^):
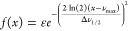
1where *x* is the energy in cm^–1^, ε is the extinction coefficient in M^–1^ cm^–1^, ν_max_ is the peak absorption energy in cm^–1^, and Δν_1/2_ is the full width at half-maximum in cm^–1^.

With the transition energy and *Δv*_1/2_ (both in cm^–1^), we can calculate the Γ parameter given by Brunschwig, Creutz, and Sutin:^22^
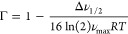
2where *R* is the ideal gas or Boltzmann constant (0.695 cm^–1^/K in these units), and *T* is the temperature in K. The fitted molar absorptivity, peak absorption energy, full width at half-maximum, and Γ for both conformations in toluene at 21 and −25 °C are presented in Table 2, and the parameters for all fits across solvents and temperatures are provided in Table S4.

**Table 2:** Gaussian Fit Parameters for **3-[Yb**_**2**_**]**^**5+**^ in Toluene at 21 and −25 °C

Isomer	Temp (°C)	ε (M^–1^ cm^–1^)	λ (nm) ν (cm^–1^)	Δν_1/2_ (cm^–1^)	Γ
**Closed**	21	144.2	721	3262	0.40
			13870		
**Closed**	–25	84.9	720	3255	0.40
			13880		
**Open**	21	42.9	591	4040	0.35
			16910		
**Open**	–25	97.1	585	3287	0.42
			17080		

D’Alessandro and Keene offer the generalization that Robin-Day Class II systems are generally weak (ε ≤ 5000 M^–1^ cm^–1^), broad (*Δv*_1/2_ ≥ 2000 cm^–1^), and exhibit greater solvent dependence than Class III systems.^21^ By these quantitative benchmarks, the Yb_2_^5+^ system described above is certainly within the bounds of Class II; however, there is a limited solvent dependence. The Γ parameter, which is proposed to measure the extent of electron delocalization,^22^ ranges from 0.2 to 0.5 depending on the conformation and temperature, with the widest range observed in diethyl ether. Brunschwig et al. provide Γ = 0.5 as a threshold for the Class II/III transition for electron delocalization; however, that value is based on *v*_*max*_ of 8000 cm^–1^ (1250 nm), and Γ increases with *v*_*max*_. Furthermore, these heuristics are calibrated for transition metal complexes with *d* orbital involvement. In the case of a 4*f*^14^ →4*f*^13^ transition, we observe much lower intensity (as is typical with other *f* orbital transitions) and a higher transition energy. As this is the first fully characterized and parametrized purely 4*f* orbital based IVCT, and there are multiple conformations available in solution, it is not yet possible to fully contextualize these parameters compared to the much more well-established transition metal IVCT transitions.

### dc Magnetometry and EPR Spectroscopy

**Figure 4:**
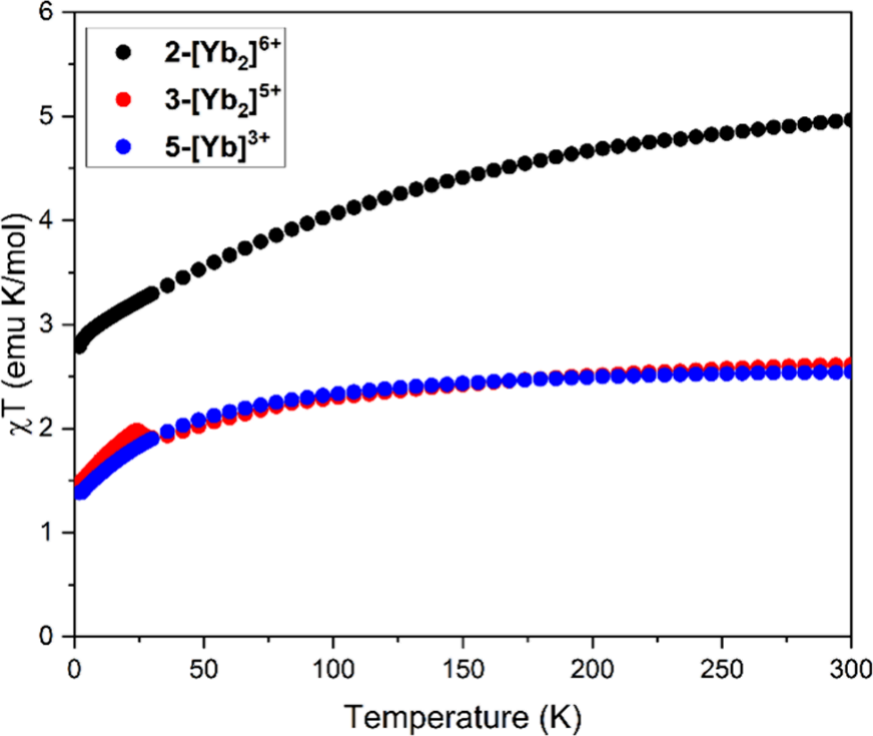
dc SQUID magnetometry variable temperature susceptibility traces for **2-[Yb**_**2**_**]**^**6+**^ (black), **3-[Yb**_**2**_**]**^**5+**^ (red), and **5-[Yb]**^**3+**^ (blue) at a 0.1 T applied field. The 300 K χT values are 4.97 (**2-[Yb**_**2**_**]**^**5+**^), 2.61 (**3-[Yb**_**2**_**]**^**5+**^), and 2.54 (**5-[Yb]**^**3+**^) emu K/mol.

dc SQUID magnetometry measurements were carried out on **2-[Yb**_**2**_**]**^**6+**^, **3-[Yb**_**2**_**]**^**5+**^, and **5-[Yb]**^**3+**^. The χ*T* vs *T* traces at 0.1 T are shown in Figure 4. The expected 300 K χ*T* value for a free Yb^3+^ ion is 2.58 emu K/mol, with observed values falling in a range of about 2.53–2.58.^42^ Compound **2-[Yb**_**2**_**]**^**6+**^ matches well with the expected value for two noninteracting Yb^3+^ ions, while compounds **3-[Yb**_**2**_**]**^**5+**^ and **5-[Yb]**^**3+**^ both match the expected value for a single Yb^3+^ ion. All three compounds exhibit a gradual decrease in χ*T* as the temperature decreases, which accelerates at lower temperature. This feature can be attributed to the thermal depopulation of crystal field states. The excellent agreement in χ*T* between **3-[Yb**_**2**_**]**^**5+**^ and **5-[Yb]**^**3+**^ is definitive evidence against any 5*d* orbital occupation in the system, confirming the mixed 4*f*^14^–4*f*^13^ occupation of the Yb^2+^ and Yb^3+^ ions, respectively. **3-[Yb**_**2**_**]**^**5+**^ was also subjected to high-pressure magnetometry to investigate whether hydrostatic pressure could induce intramolecular coupling, 5*d* occupation for Yb^2+^, or bonding between the Yb^2+^ and Yb^3+^ ions; however, no significant pressure dependence was observed (see SI for methodological details and Figures S8–S10 for plots of pressure dependent magnetic susceptibility and saturation).

**Figure 5:**
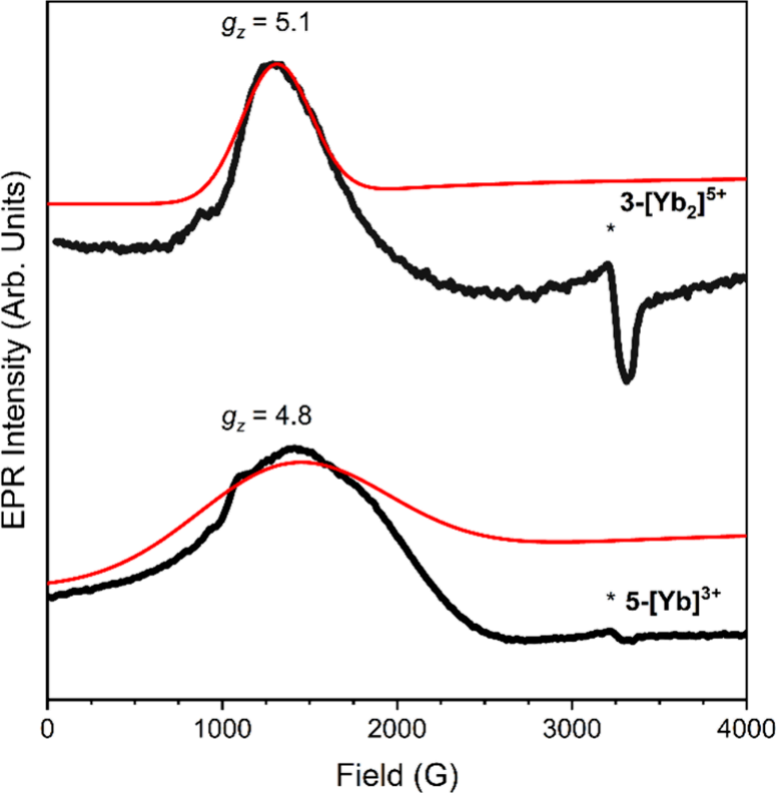
EPR spectra of **3-[Yb**_**2**_**]**^**5+**^ and **5-[Yb]**^**3+**^ are colored black along with simulations to determine the *g* value in red.

Continuous wave X-band EPR spectroscopy of **3-[Yb**_**2**_**]**^**5+**^ and **5-[Yb]**^**3+**^ are consistent with the dc susceptibility analysis and are shown in Figure 5. The spectra of both **3-[Yb**_**2**_**]**^**5+**^ and **5-[Yb]**^**3+**^ show single features suggestive of an axial *g*-tensor with *g*_∥_ ≫ *g*_⊥_. These spectra can be simulated using *g*_∥_ = 5.1 and 4.8, respectively, with *g*_⊥_ below the minimum of ∼0.67 determined by the maximum available magnetic field.

X-ray absorption near edge spectroscopy (XANES) provides further support for the Yb^2+^/Yb^3+^ mixed oxidation state. Compounds **1-[Yb**_**2**_**]**^**6+**^, **2-[Yb**_**2**_**]**^**6+**^, and **5-[Yb]**^**3+**^ all display a single feature at the L_3_ edge at 8945–8947 eV attributed to the 2*p* →5*d* transition in Yb^3+^. Compound **3-[Yb**_**2**_**]**^**5+**^ displays the same 8945 eV peak, as well as a second peak at 8937 eV. Peak energy values determined by both fitting and inflection point are given in Table 3. The energy difference of ∼8 eV is consistent for the Ln^2+^ and Ln^3+^ oxidation states for purely 4*f*^n^ configurations,^43^ with the lower energy peak attributed to Yb^2+^ while the higher energy peak corresponds to Yb^3+^. Figure S11 shows the spectra along with the fits used to determine the peak positions. Integrated peak intensities for **3-[Yb**_**2**_**]**^**5+**^, however, cannot be interpreted in detail because the compound undergoes photoinduced oxidation over the course of the measurement. Figure S12 demonstrates the decrease in the Yb^2+^ feature intensity and increase in the Yb^3+^ feature intensity over successive scans, despite 10 K helium cryoprotection. Similar photoinduced decomposition has been noted in other Ln^2+^ systems.^43^

**Table 3:** XANES Peak Energies Determined by Both Peak Fitting and Inflection Points

Compound	Fitted Peak Energies (eV)	Inflection Point Peak Energies (eV)
**1-[Yb**_**2**_**]**^**6+**^	8946.70(7)	8944.0
**2-[Yb**_**2**_**]**^**6+**^	8946.40(4)	8944.0
**3-[Yb**_**2**_**]**^**5+**^	8937.34(8)	8936.0
	8945.37(7)	8943.5
**5-[Yb]**^**3+**^	8946.27(5)	8943.9

### Theoretical Analysis

**Figure 6:**
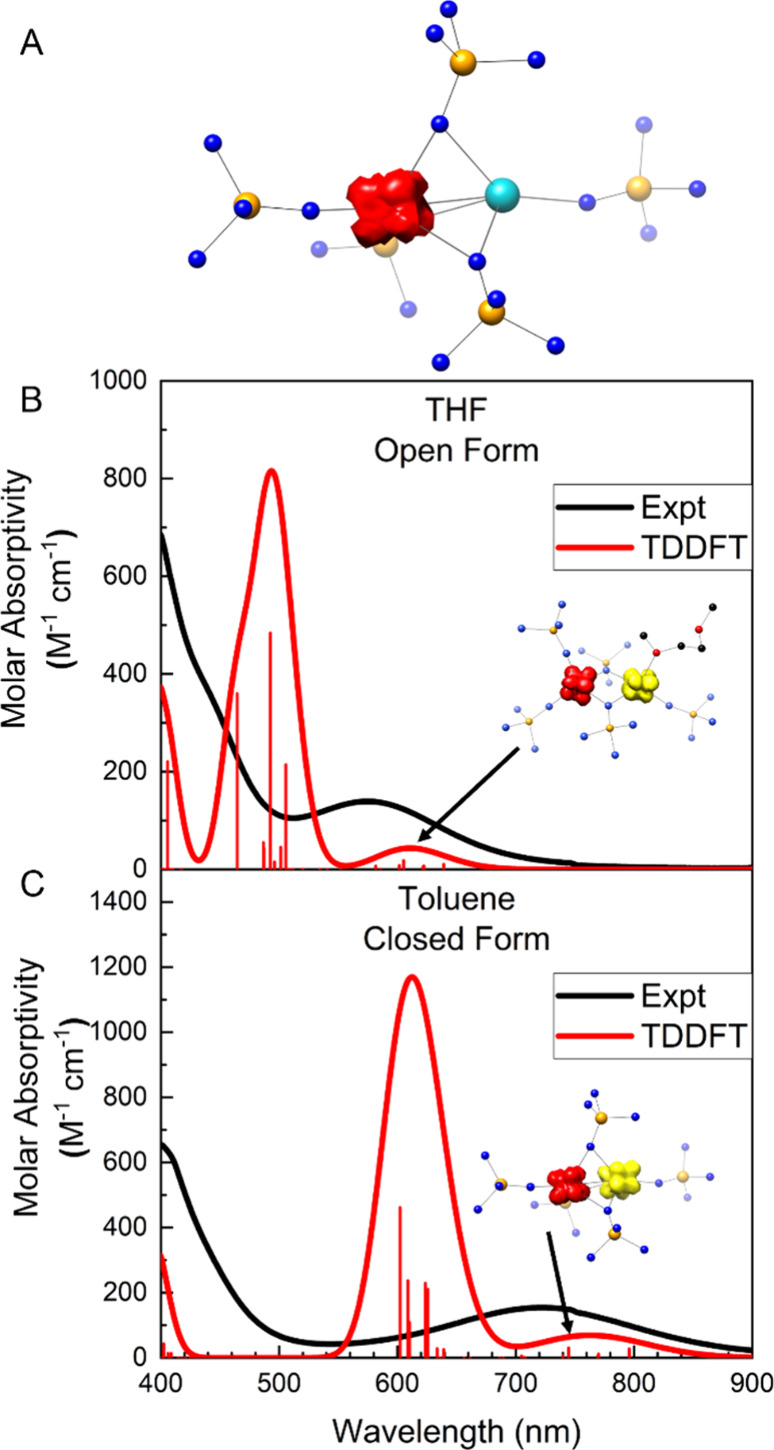
(A) DFT-calculated spin density for **3-[Yb**_**2**_**]**^**5+**^. (B) TDDFT spectral simulations for **4-[Yb**_**2**_**]**^**5+**^ overlaid with the spectrum of **3-[Yb**_**2**_**]**^**5+**^ in THF, resulting in the open configuration. Inset is an electron density difference map, with yellow showing the donor orbitals and red showing the acceptor orbitals. (C) TDDFT spectral simulations for **3-[Yb**_**2**_**]**^**5+**^ overlaid with the spectrum of **3-[Yb**_**2**_**]**^**5+**^ in toluene, resulting in the closed configuration. Inset is an electron density difference map, with yellow showing the donor orbitals and red showing the acceptor orbitals.

Electronic structure calculations and simulations of experimental data were carried out for **1-[Yb**_**2**_**]**^**6+**^, **3-[Yb**_**2**_**]**^**5+**^, **4-[Yb**_**2**_**]**^**5+**^, and **5-[Yb]**^**3+**^. A combination of density functional theory (DFT) and state averaged complete active space self-consistent field with N-electron valence perturbation theory including spin–orbit coupling (SA-CASSCF/NEVPT2 + SOC) calculations were performed. DFT, including time-dependent DFT (TDDFT), is less accurate for modeling spin orbit coupling in lanthanides, but the CAS methods are computationally expensive and were therefore limited to the 4*f* orbitals. A DFT spin density calculation for both mixed-valence **3-[Yb**_**2**_**]**^**5+**^ (Figure 6A) and **4-[Yb**_**2**_**]**^**5+**^ show complete localization of spin and unambiguous Yb^2+^ and Yb^3+^ ions, consistent with the crystallographic bond distances in these compounds.

Across all four compounds, TDDFT predicts the *f* → *f* transitions at much lower energy than is observed. The CAS methods, however, accurately predict the energy of these transitions (Figure S14). Conversely, the CAS methods are limited to 4*f* electrons and orbitals, and therefore do not model the high energy charge transfer features, which TDDFT methods identify as LMCT. In the mixed-valence species, **3-[Yb**_**2**_**]**^**5+**^ and the solvated open-form **4-[Yb**_**2**_**]**^**5+**^, TDDFT methods predict intermediate energy and intensity features, attributable to IVCT (Figure 6 B, 6C). The insets of Figure 6 B and C show the calculated electron density difference maps for the IVCT calculated in both the closed and open configurations. In both cases, yellow represents donor orbital density, and red represents acceptor orbital density. These densities are located entirely on the two Yb ions with no meaningful density on the bridging ligands. Therefore, the bridging ligands are not offering an electronic pathway for the IVCT. Instead, the IVCT occurs directly between 4f orbitals, and the ligands are only involved insofar as to enforce the short Yb···Yb distance.

Both the energy and intensity of the IVCT are overestimated by TDDFT compared to the observed features. Weak IVCT bands are also predicted by CAS. Interestingly, both CAS and DFT methods predict a shift to higher energy for the IVCT in **4-[Yb**_**2**_**]**^**5+**^ compared to **3-[Yb**_**2**_**]**^**5+**^, matching the trend observed experimentally. Finally, the higher energy feature observed at 380 nm in **3-[Yb**_**2**_**]**^**5+**^ is predicted by TDDFT to occur at a much lower energy: 612 nm for **3-[Yb**_**2**_**]**^**5+**^ and 493 nm for **4-[Yb**_**2**_**]**^**5+**^. It is possible that these predicted transitions are instead overlapping with the observed IVCT and do not correspond with the 380 nm feature; however, the 4*f* → 5*d* is often observed in purely Yb^2+^ systems at higher energy.^40,41^ This transition is also predicted to donate from the 4*f* orbitals to a mixture of 5*f*, 6*s*, and 6*p* orbitals, despite the typical assignment of similar features as simply 4*f* → 5*d*.

## Discussion

To date, lanthanide-based IVCT transitions have been limited to extended solid materials. There are only a few examples of mixed-valence molecular lanthanide complexes, but even those with shorter Ln···Ln distances have not had IVCT transitions reported. Where spectra have been reported for traditional 4*f*^n^ lanthanides (Sm^2+^, Eu^2+^) the observed features can be attributed to 4*f* → 5*d* transitions observed for Ln^2+^ ions individually.^6,8^ Formally mixed-valence systems with divalent lanthanides that possess mixed 4*f*^n-1^5*d*^1^ configurations conversely have recently been shown to exhibit metal–metal bonding. While the σ and σ* orbitals of 5*d*_z^2^_ parentage exhibit a σ → σ* transition that is visually indistinguishable from IVCT (and is often referred to as a Robin-Day Class III IVCT), such systems are more accurately described as *intermediate* valence (i.e., Ln^2.5+^), and the σ → σ* transition has neither intervalence nor charge transfer character. Therefore, to demonstrate IVCT, one must identify the spectroscopic transition while ruling out alternative electronic origins of the transition.

In the case of **3-[Yb**_**2**_**]**^**5+**^, the assignment of mixed-valence readily follows from the structural data. The five imidophosphorane ligands are closed shells, requiring an Yb_2_^5+^ oxidation state to achieve a charge-neutral complex. The lack of crystallographic equivalence between the two ytterbium ions and the significantly increased Yb–N distances for one ion are unambiguous indicators of Yb^2+^ and Yb^3+^ ions in the structure. Consequently, the features in the electronic absorbance spectrum at ∼720 or ∼575 nm, depending on solvent and temperature, are candidates for IVCT absorption.

Gould, Long, and co-workers recently demonstrated the first Ln–Ln bond, achieved through reduction of a Ln_2_^6+^ dimeric species to Ln_2_^5+^. The resulting bond, a two-center, one-electron bond with a formal bond order of 1/2, has a dramatic impact on the magnetic ground state of the molecule. When unpaired 4*f* electrons are present, a double-orthogonality coupling mechanism, as described by Chipman and Berry in transition metal systems,^25^ results in an extraordinarily large ferromagnetic coupling between the ions. **3-[Yb**_**2**_**]**^**5+**^ exhibits no ferromagnetic coupling, instead displaying magnetism consistent with noninteracting Yb^3+^ (*J* = 7/2) and Yb^2+^ (diamagnetic) ions. Therefore, we can confidently assign the visible spectrum feature of **3-[Yb**_**2**_**]**^**5+**^ to an IVCT transition.

It is informative to consider why previously reported mixed-valence lanthanide complexes do not exhibit observable IVCT transitions. Given that both the closed and the open configurations demonstrate IVCT, we cannot correlate the shorter internuclear distance in **3-[Yb**_**2**_**]**^**5+**^ to the presence or intensity of the IVCT feature. Other mixed-valence compounds with close Ln···Ln distances either do not report UV/vis/NIR spectra,^4,5,7,9−11^ or the only features in the UV/visible region can be attributed to the individual Ln^2+^ and Ln^3+^ ions, and NIR spectra are not reported.^6,8^ It is therefore not possible to rule out unreported IVCT features in previously reported compounds.

## Conclusion

A series of ytterbium-containing compounds, including two conformations of a Yb_2_^5+^ mixed-valence complex, were prepared and characterized. Bonding metrics determined by single crystal X-ray diffraction suggest localization of Yb^2+^ and Yb^3+^ character in the mixed-valence complexes, as well as an exceptionally short Yb···Yb distance of 2.9507(8) Å in the nonsolvated **3-[Yb**_**2**_**]**^**5+**^. An electronic absorption feature was identified and assigned as IVCT based on structural metrics, magnetic properties, and computational analysis. Both the variable temperature magnetic susceptibility and the theoretical model suggest negligible interaction between Yb^2+^ and Yb^3+^ ions, despite the short distance.

The assignment of an IVCT transition in a molecular mixed-valence lanthanide complex is the first of its kind. Previously reported mixed-valence complexes either were extended solids or did not present assignable IVCT transitions. The ability of a Yb_2_^5+^ complex to demonstrate electronic transitions due to 4*f*^13 2^F^7/2^ → ^2^F_5/2_, 4*f*^14^ 4*f* → 5*d*, and 4*f*^14^ → 4*f*^13^ IVCT simultaneously is a unique result of the combination of the accessible near-valence orbitals in Yb^3+^ and Yb^2+^ and the generation of the IVCT in the mixed-valence complex. This combination of electronic absorption features presents the opportunity to consider potential technical applications derived from the control of these electronic excitations.

As the collection of mixed-valence and metal–metal bonded lanthanide and actinide complexes continues to grow, it is important to recognize that fundamentally different electronic structures can give rise to similar optical transitions. The results and analyses presented here show how a combination of structural analysis, spectroscopy, and magnetometry enables the definitive assignment of the underlying electronic structure. These tools offer a clear path forward in the development of both metal–metal bonding and mixed-valence IVCT transitions in complexes of *f*-block elements.

## Supporting Information Available

Detailed experimental procedures, crystallographic details, magnetic measurements, XANES spectra, computational details, discussion of metal–metal distances, NMR spectra, and fits of spectral data (PDF)
