# Supplementary material for: Intervalence Charge Transfer in Nonbonding, Mixed-Valence, Homobimetallic Ytterbium Complexes
Source: J Am Chem Soc. 2024 Feb 19;146(8):5560–8. doi: 10.1021/jacs.3c13906 (PMC10910554; doi:10.1021/jacs.3c13906)
Supplement: Supplementary file 1 — ja3c13906_si_001.pdf [file ja3c13906_si_001.pdf]

Supporting Information for:

Intervalence Charge Transfer in Nonbonding Mixed-Valence, Homo-bimetallic Ytterbium Complexes

Michael D. Roy[a†], Thaige P. Gompa[a†], Samuel M. Greer[b], Ningxin Jiang[a], Lila S. Nassar[c], Alexander Steiner[d], John Bacsa[a], Benjamin W. Stein[b], and Henry S. La Pierre[a,e,f]*

[a]*School of Chemistry and Biochemistry, Georgia Institute of Technology, Atlanta, Georgia 30332-0400, United States*

[b]*Los Alamos National Laboratory, Los Alamos, New Mexico 87545, United States.*

[c]*School of Physics, Georgia Institute of Technology, Atlanta, Georgia 30332-0400, United States*

[d]*Department of Chemistry, University of Liverpool, Liverpool L69 7Zd, United Kingdom*

[e]*Nuclear and Radiological Engineering Program, Georgia Institute of Technology, Atlanta, Georgia 30332-0400, United States*

[f]*Physical Sciences Division, Pacific Northwest National Laboratory, Richland, Washington 99352, United States*

†*These authors contributed equally to this work.*

**To whom correspondence should be addressed.*

Contents

General Considerations 3

Synthetic Procedures 3

X-Ray Crystallography 5

Electrochemistry 6

Magnetic Measurements 7

Yb L_3-edge X-ray Absorption Near Edge Spectroscopy (XANES) 13

Quantum Chemical Calculations 15

Discussion of Metal-metal Distance Comparisons 17

NMR Spectra 19

UV/vis Spectral Fits 24

- Diethyl Ether 25
- Hexanes 28
- Toluene 30

Crystallographic Information 33

- 1-$[Yb_2]^{6+}$ 33
- 2-$[Yb_2]^{6+}$ 35
- 3-$[Yb_2]^{5+}$ 37
- 4-$[Yb_2]^{5+}$ 39
- 5-$[Yb]^{3+}$ 41

Example Calculation Input Files 43

References 47

General Considerations

Unless otherwise noted, all reagents were obtained from commercial suppliers. The syntheses and manipulations were conducted under argon with exclusion of oxygen and water using Schlenk techniques or in an inert atmosphere box (Vigor) under a dinitrogen (<0.1 ppm O_2/H_2O) atmosphere. All glassware was stored in an oven over-night (>8 h) at a temperature of ca. 160˚C. $YbI_3(THF)_{3.5}$[1] and $KNP(pip)_3$,[2] were prepared by literature methods. KC_8 was prepared by melting potassium metal over graphite in a dinitrogen glovebox. Celite and molecular sieves were dried under vacuum at a temperature >250˚C for a minimum of 24 h. C_6D_6 was degassed, stored over 3 Å molecular sieves for at least 48 hours, then vacuum-transferred from sodium/benzophenone prior to use. THF-d_8 was degassed, dried over sodium for at least one week, and then vacuum-transferred and stored over 3 Å molecular sieves. All non-deuterated solvents were purged with UHP-grade argon (Airgas) and passed through columns containing Q-5 and molecular sieves in a solvent purification system (JC Meyer Solvent Systems). All solvents in the glovebox were stored in bottles over 3 Å molecular sieves. NMR spectra were obtained on a Bruker Advance III 400 MHz spectrometer at 298 K, unless otherwise noted. NMR chemical shifts are reported in δ, parts per million and referenced to the residual ^{1}H resonances of the deuterated solvent. To account for low signal to noise, ^{1}H and $^{13}C\{^1H\}$ NMR spectra were treated with 5.0 Hz exponential line broadening, and $^{31}P\{^1H\}$ NMR spectra were treated with 10.0 Hz exponential line broadening. All complexes reported here are paramagnetic and dynamic in solution and have NMR spectra incongruent with their crystallographically determined structures. They are therefore tabulated without assignment and full spectra are included for identification. Attempts to find a temperature regime that leads to either full coalescence (averaged structure) or decoalescence were unsuccessful due to solvent compatibility with the complex and/or the accessible temperature window (freezing/boiling temperature) of the NMR solvent. Infrared (IR) samples were taken on a Bruker ALPHA FTIR spectrometer with ATR attachment from 400 to 4000 cm^{-1}. The peaks are listed in wavenumber [cm^{-1}] and intensity by using the following abbreviations: vw (very weak); w (weak); m (medium); s (strong); vs (very strong); br (broad). UV/vis/NIR spectroscopy was performed in PTFE-valve sealed quartz cuvettes with a 1 cm path length on a Hitachi UH4150 UV–vis–NIR scanning spectrophotometer between 2500 and 240 nm. Elemental analyses were determined at Robertson Microlit Laboratories (Ledgewood, NJ) and the University of California Berkeley Microanalytical Facility (Berkeley, CA). X-band EPR spectra were recorded using a commercial Bruker E680 X-band spectrometer. The sample temperature was controlled using an Oxford Instruments CF935 helium flow cryostat and ITC503 temperature controller. Measurements were performed on polycrystalline samples and spectral simulations were carried out using Easyspin.[3]

Synthetic Procedures

Synthesis of 1-[Yb_2]$^{6+}$. Inside a glovebox, $YbI_3(THF)_{3.5}$ (0.230 g, 0.285 mmol) was added to a 20 mL scintillation vial charged with a stir bar and 1 mL of THF. K[$NP(pip)_3$] (0.302 g, 0.857 mmol, 3.0 eq.) was added to the stirring mixture as a solution in 4 mL of THF. The reaction mixture was stirred overnight. The mixture was filtered through a fine porosity frit packed with Celite. The filtrate was concentrated *in vacuo* to give a tan solid. The residue was triturated three times with 1 mL of *n*-pentane and then taken up in 8 mL of toluene and filtered through a pipet filter packed with Celite and glass filter paper. The solution was concentrated *in vacuo* and placed inside a −35 °C freezer overnight, during which time colorless crystals were obtained (0.491 g, 81%). ^{1}H NMR

(400 MHz, THF-d_8) δ 56.45, 15.41, 11.97, 8.87, 7.11, 2.95, 1.42, -0.69, -2.71, -9.21, -12.28, -36.34 ppm. $^{31}P\{^1H\}$ NMR (162 MHz, THF-d_8): δ 33.55, -102.12, -208.79, -253.92 ppm. No ^{13}C NMR peaks were observed. IR: ν [cm^{-1}] = 1260 (m), 1208 (w), 1172 (s), 1103 (m), 1063 (m), 1027 (m), 938 (s), 800 (m), 573 (m), 488 (w). Elemental analysis for $C_{90}H_{180}N_{24}P_6Yb_2{\bullet}C_7H_8$ found (calculated): C, 52.00 (52.42), H, 8.58 (8.53), N, 15.82 (15.12). Analytically pure material was prepared by crystallization from a saturated toluene solution at -35 °C, followed by decantation and rinsing with pentane. XRD quality crystals were grown from a saturated toluene solution at -35 °C.

Synthesis of 2-[Yb_2]$^{6+}$. Inside a glovebox, $YbI_3(THF)_{3.5}$ (0.300 g, 0.372 mmol) was added to a 20 mL scintillation vial charged with a stir bar and 1 mL of THF. K[NP(pip)$_3$] (0.300 g, 0.857 mmol, 2.3 eq.) was dissolved separately in 5mL of THF. 2 mL of the K[NP(pip)$_3$] solution was added directly to the stirring slurry of $YbI_3(THF)_{3.5}$. The remaining 3 mL of K[NP(pip)$_3$] solution was added dropwise over 10 minutes under vigorous stirring. The reaction mixture was stirred overnight. The mixture was filtered through a fine porosity frit packed with Celite. The filtrate was concentrated *in vacuo* to give a yellow solid. The residue was triturated three times with 1 mL of *n*-pentane and then taken up in 5 mL of 1,2-dimethoxyethane and filtered through a pipet filter packed with Celite and glass filter paper. The dark yellow/orange solution was placed inside a −35 °C freezer overnight, during which time yellow crystals were obtained (0.561 g, 83% in ligand, 76% in metal). ^1H NMR (400 MHz, C_6D_6) δ 46.14, 28.70, 26.87, 20.95, 17.8, 12.69, 6.81, 4.18, 1.77, 1.19, 0.85, 0.17, -1.11, -1.93, -5.06, -7.78, -9.20, -10.35, -10.97, -11.29, -12.46, -15.64, -18.06, -27.18, -46.74 ppm. $^{31}P\{^1H\}$ NMR (162 MHz, C_6D_6): δ 151.51, -142.16 ppm. No ^{13}C NMR peaks were observed. IR: ν [cm^{-1}] = 1260 (m), 1208 (w), 1172 (s), 1103 (m), 1063 (m), 1027 (m), 938 (s), 800 (m), 573 (m), 488 (w). Elemental analysis for $C_{75}H_{150}N_{20}P_5IYb_2$ found (calculated): C, 46.13 (45.96), H, 7.77 (7.71), N, 14.39 (14.29). Analytically pure material was prepared by crystallization via evaporation of a concentrated solution in *n*-pentane. XRD quality crystals were grown through evaporation of 1,2-dimethoxyethane solution at room temperature.

Synthesis of 3-[Yb_2]$^{5+}$. Inside a glovebox, **2-[Yb_2]$^{6+}$** (0.400 g, 0.204 mmol) was added to a 20 mL scintillation vial charged with a glass stir bar and 4 mL of diethyl ether. Potassium graphite (KC_8) (0.028 g, 0.204 mmol, 1.0 eq.) was added to the stirring mixture as the solution in 4 mL of diethyl ether. The reaction mixture was stirred for 2h. The mixture was filtered through a fine porosity frit packed with Celite. The filtrate was concentrated *in vacuo* to give green solid. The residue was triturated three times with 1 mL of *n*-pentane and then taken up in 5 mL of n-hexanes and filtered through a pipet filter packed with Celite and glass filter paper. The solution is stored overnight inside a −35 °C freezer. The next day, purple solid has precipitated from the solution. The solution is filtered through a pipet filter packed with Celite and glass filter paper. The filtrate was concentrated *in vacuo* to give green solid. The solid is redissolved in 1-2 mL of n-pentane. The solution was concentrated *in vacuo* and placed inside a −35 °C freezer in an evaporation setup, during which time green crystals were obtained (0.220 g, 59%). ^1H NMR (400 MHz, C_6D_6) δ 19.34, 12.26, 3.00, 1.36, 0.28, -2.71, -3.08, -3.32, -3.91, -5.11, -5.70, -9.98, -11.26, -23.30 ppm. The peaks at 3.00 and 1.36 ppm integrate in a 4:6 ratio analogous to the free ligand and may be attributable to the ligand bound terminally to the Yb^{2+} ion. $^{31}P\{^1H\}$ NMR (162 MHz, C_6D_6): δ 34.51, -0.67 ppm. No ^{13}C NMR peaks were observed. IR: ν [cm^{-1}] = 1264 (m), 1208 (w), 1100 (m), 1030 (m), 1024 (m), 938 (s), 800 (m), 725 (w), 573 (m), 488 (w). Elemental analysis for

$C_{75}H_{150}N_{20}P_5Yb_2$ found (calculated): C, 48.13 (49.14), H, 8.00 (8.25), N, 15.14 (15.28). XRD quality crystals were grown from an evaporating saturated *n*-pentane solution at -35 °C.

Crystallographic Identification of 4-[Yb$_2$]$^{5+}$. Recrystallization of **3-[Yb$_2$]$^{5+}$** from 1,2-dimethoxyethane yielded crystals identified as **4-[Yb$_2$]$^{5+}$** by X-ray crystallography.

Synthesis of 5-[Yb]$^{3+}$. Inside a glovebox, $YbI_3(THF)_{3.5}$ (0.230 g, 0.285 mmol) was added to a 20 mL scintillation vial charged with a stir bar and 1 mL of THF. K[NP(pip)$_3$] (0.394 g, 1.14 mmol, 4.0 eq.) was added to the stirring mixture as a solution in 5 mL of THF. The reaction mixture was stirred overnight. The mixture was filtered through a fine porosity frit packed with Celite. The filtrate was concentrated *in vacuo* to give a tan solid. The residue was triturated three times with 1 mL of *n*-pentane. Dry [2.2.2]Cryptand (0.107 g, 0.285 mmol, 1.0 eq.) was dissolved in 6 mL of DME. This solution is added to the tan solid to dissolve it. The mixture was filtered through a fine porosity frit packed with Celite. The solution was concentrated *in vacuo* and placed inside a −35 °C freezer overnight, during which time clear crystals were obtained (0.360 g, 71%). ^{1}H NMR (400 MHz, C_6D_6) δ 12.93, 9.02, 4.52, 4.17, 3.27, 3.06, 2.40, 2.32, 1.89, 1.69, 1.14, 0.84, 0.13 ppm. ^{31}P{^{1}H} NMR (162 MHz, C_6D_6): δ 87.03, -131.09 ppm. ^{13}C{^{1}H} NMR (101 MHz, C_6D_6) δ 52.04, 29.93, 28.19 ppm. IR: ν [cm^{-1}] = 1438 (m), 1355 (m), 1322 (m), 1258 (w), 1192 (vs), 1150 (m), 1105 (s), 1080 (w), 1043 (s), 1027 (s), 923 (s), 852 (m), 830 (m), 752 (w), 696 (s), 664 (m), 559 (s), 550 (s), 479 (s), 468 (s). Analytically pure material was prepared by washing the product with *n*-pentane, recrystallizing from 1,2-dimethoxyethane, decanting the mother liquor, and rinsing again with *n*-pentane. Elemental analysis for $C_{78}H_{156}N_{18}KO_6P_4Yb$ found (calculated): C, 52.39 (52.68), H, 8.64 (8.84), N, 14.20 (14.18). XRD quality crystals were grown from a saturated DME solution at -35 °C.

X-Ray Crystallography

Crystals suitable for X-ray diffraction were covered in paratone oil in a glove box and transferred to the diffractometer in a 20 mL capped vial. Crystals were mounted on a loop with paratone oil on a Bruker D8 VENTURE diffractometer. The crystals were cooled and kept at T = 100(2) K during data collections. The structures were solved with the ShelXT structure solution program using the Intrinsic Phasing solution method and by using Olex2 as the graphical interface.[4, 5] The model was refined with version 2014/7 of XL using Least Squares minimization.[6] Figures were generated using Mercury.[7]

Additional refinement details for each compound are as follows. For **1-[Yb$_2$]$^{6+}$**, two disordered molecules of THF are present in the lattice. **4-[Yb$_2$]$^{5+}$** was modeled as a two-component twin with a two-fold rotation about [1 -1 0]. Additionally, there was found to be a minor occupation of iodide in place of the coordinated DME. The occupation was refined freely and then fixed to 0.15. In **2-[Yb$_2$]$^{6+}$**, **3-[Yb$_2$]$^{5+}$**, **4-[Yb$_2$]$^{5+}$**, and **5-[Yb]$^{3+}$**, disorder in the piperidinyl rings was modeled using the fewest constraints and restraints possible to afford a reasonable solution. No disorder modeling was necessary for the ytterbium ions or their nearest neighbors.

Electrochemistry

Electrochemical measurements were performed in a glove box using a Pine WaveDriver 20 potentiostat and a three-electrode cell. Glassy carbon encased in PCTFE (poly(chlorotrifluoroethylene)) was used as the working electrode, bare platinum wire was used as the counter electrode, and polished silver wire in a fritted capillary was used as a pseudo-reference electrode. The electrolyte solution (0.05 M triply-recrystallized tetra-n-butylammonium tetraphenylborate in THF) was prepared freshly before experiments. Electrical potential was referenced against a decamethylferrocene internal standard added for the final scan. This potential was normalized to the reduction of ferrocenium using the redox couple of decamethylferrocene at -0.50 V vs $Fc^{+/0}$ in the same electrolyte solution.[8]

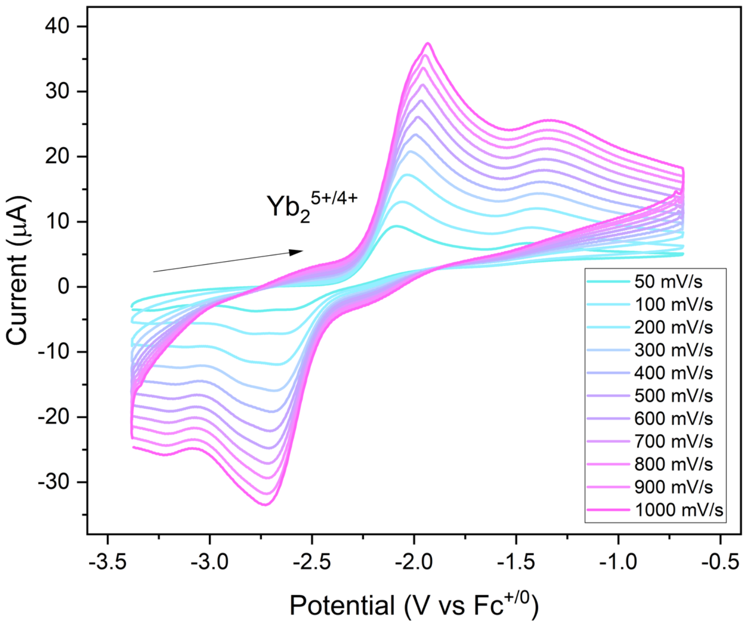

Figure S1. Scan rate dependence of the $[Yb_2]^{5+/4+}$ couple (2.47 mM **3-$[Yb_2]^{5+}$** in THF; 0.05 M $TBABPh_4$). The second of three scans (segments three and four of six) is shown. The anodic feature at ~ -1.5 V only occurs following the cathodic feature at ~ -2.75 V, suggesting chemical speciation or decomposition following electrochemical reduction.

Table S1. Scan rate dependence peak currents and potential differences for **3-[Yb$_2$]$^{5+}$**.

Scan Rate (mV/s)	Square Root of Scan Rate (mV/s)$^{1/2}$	**I_{pc}** (μA)	**I_{pa}** (μA)	$\lvert I_{pc}/I_{pa}\rvert$	ΔE_p (mV)
**50**	7.07	-3.73	9.32	0.400	684
100	10	-7.22	13.1	0.552	714
200	14.1	-11.9	17.2	0.694	643
300	17.3	-16.0	20.8	0.768	661
400	20	-19.2	23.4	0.821	702
500	22.4	-22.2	26.1	0.850	725
600	24.5	-24.8	28.6	0.868	744
700	26.5	-27.1	31.0	0.874	755
800	28.3	-29.4	33.6	0.874	767
900	30	-31.8	35.6	0.894	771
1000	31.6	-33.5	37.4	0.894	795

The peak cathodic and anodic currents (I_{pc} and I_{pa}) for the [Yb$_2$]$^{5+/4+}$ couple are linear with respect to the square root of the scan rate, consistent with the Randles-Sevcik equation for freely diffusing analyte.[9] The ratio of peak currents is less than 1, but increases with faster scan rate, implying that this redox event is chemically quasi-reversible. The large separation (~700 mV) in peak potentials indicates an electrochemically irreversible process due to slow electron transfer kinetics. Such slow electron transfer rates are at least partly due to the lower dielectric constant of THF compared to acetonitrile as well as the lower electrolyte concentration (0.05 M compared to a typical 0.1 M). In combination with the anodic feature at ~ -1.5 V in Figure S1, these reversibility metrics suggest a chemical process following reduction that occurs at a competitive rate to the electron transfer.

Magnetic Measurements

Ambient pressure magnetic measurements were performed on a Quantum Design MPMS3 magnetometer. Inside of a glovebox, powdered samples were loaded inside of the shelved quartz tubes along with a measured amount of eicosane. The tops of the tubes were affixed to an Ultra Torr Swagelok adaptor. This was transported from the glovebox to a Schlenk line where it was sealed using a O_2/H_2 torch while the sample was under vacuum. The eicosane was then melted by placing the sealed tube in warm water, fixing the sample in place to prevent torquing under an applied magnetic field. The sealed tubes were wrapped with 1-2 rounds of PTFE tape around the top of the tube and pressed into plastic straws, which were then loaded into the instrument. Diamagnetic corrections for the eicosane and complexes were performed using Pascal's constants and are tabulated in table S2.[10] The background signal of the quartz tube is negligible compared to the sample signal and was not considered in the data analyses.

Table S2. Diamagnetic corrections for **2-[Yb$_2$]$^{6+}$**, **3-[Yb$_2$]$^{5+}$**, **5-[Yb]$^{3+}$**, and eicosane.

Compound	Diamagnetic Correction
**2-[Yb$_2$]$^{6+}$**	-1224.60*10^{-6} emu/mol
**3-[Yb$_2$]$^{5+}$**	-1174.00*10^{-6} emu/mol
**5-[Yb]$^{3+}$**	-1193.92*10^{-6} emu/mol
Eicosane	-8.6*10^{-10} emu/mg

High pressure magnetometry was conducted using the commercially available HMD pressure cell from Quantum Design. In a glovebox, crystalline **3-[Yb$_2$]$^{5+}$** was ground to a powder and added to the PTFE sample holder, followed by Daphne 7373 oil as a hydrostatic medium. The masses of sample and oil were determined by difference, and care was taken to minimize loss of material when mixing into a uniform suspension. After assembling the pressure cell, the uncompressed length was recorded. Following initial magnetic measurements, the pressure cell was compressed by 1.02 mm, 1.50 mm, and 2.00 mm relative to its initial length and magnetic measurements were recorded at each step. The pressure corresponding to each compression was determined using the calibration curve provided with the pressure cell. The background contribution of the pressure cell was determined by performing the same magnetic measurements on the empty, uncompressed cell.

SQUIDLab was used to import and process the background subtractions.[11] The raw background and sample data were processed with the following SQUIDLab parameters: no smoothing or drift subtraction, X-centering (which is the z-axis along which the sample moves in the instrument), default "pchipinterp" fitting, and averaging of consecutive scans to accommodate up and down scans. Each processed voltage scan for the background was subtracted from the corresponding sample data and the result was fit using the Levenberg-Marquardt algorithm with an experimentally determined instrument-specific calibration factor of -6.34•10^{-7}. Variable field magnetization data were plotted directly. Variable temperature susceptibility data were processed as the standard samples, neglecting the diamagnetic contribution of the Daphne oil.

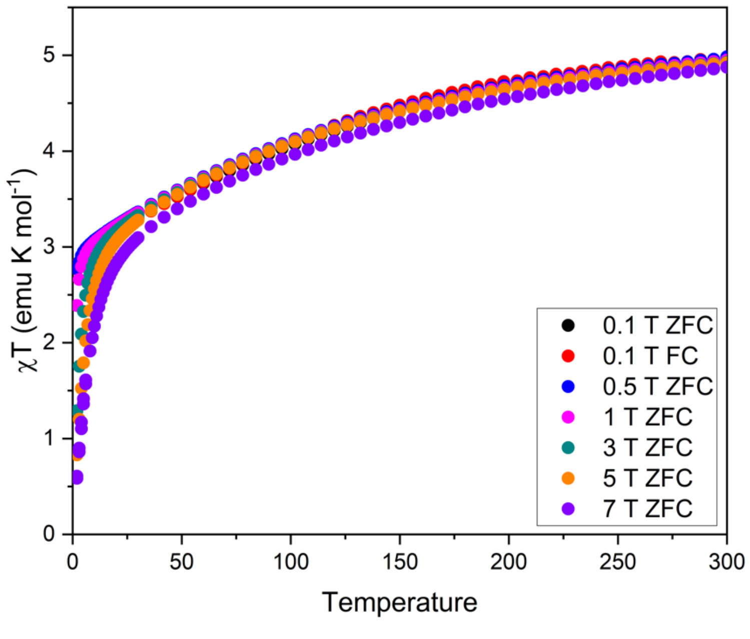

Figure S2. Variable field χT vs T plots for **2-[Yb$_2$]$^{6+}$**. The typically observed room temperature χT for two Yb^{3+} ions is 5.0-5.2 emu K mol^{-1}.

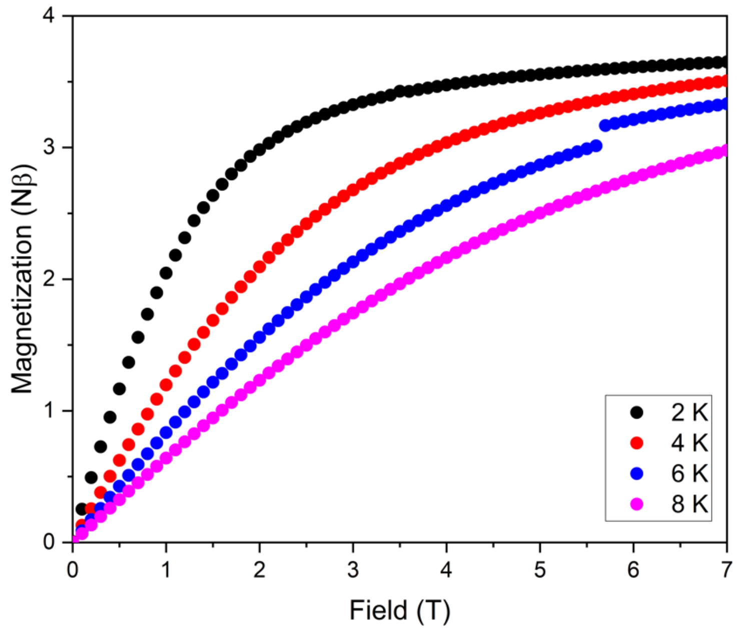

Figure S3. Variable field magnetization plots for **2-[Yb$_2$]$^{6+}$**. The discontinuity in the 6 K data around 6 T is due to an instrument error during the data collection sequence.

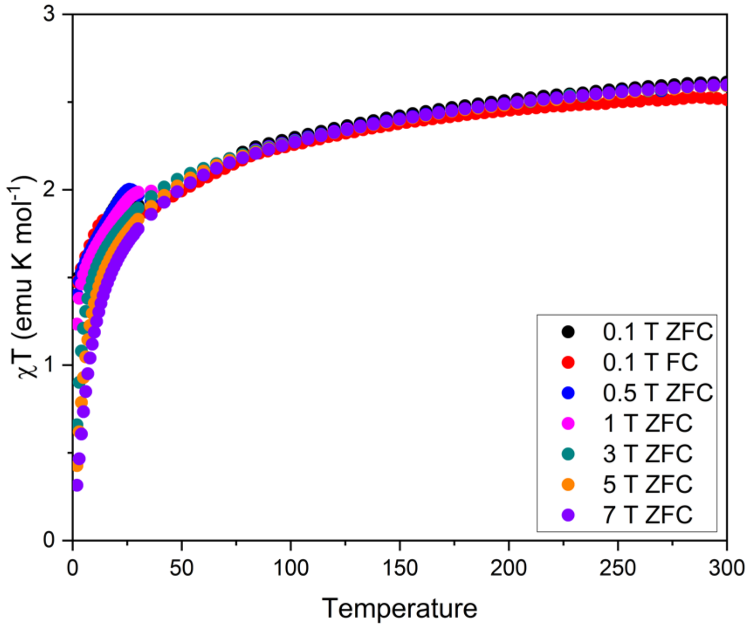

Figure S4. Variable field χT vs T plots for **3-[Yb$_2$]$^{5+}$**. The typically observed room temperature χT for one Yb^{3+} ion is 2.5-2.6 emu K mol^{-1}.

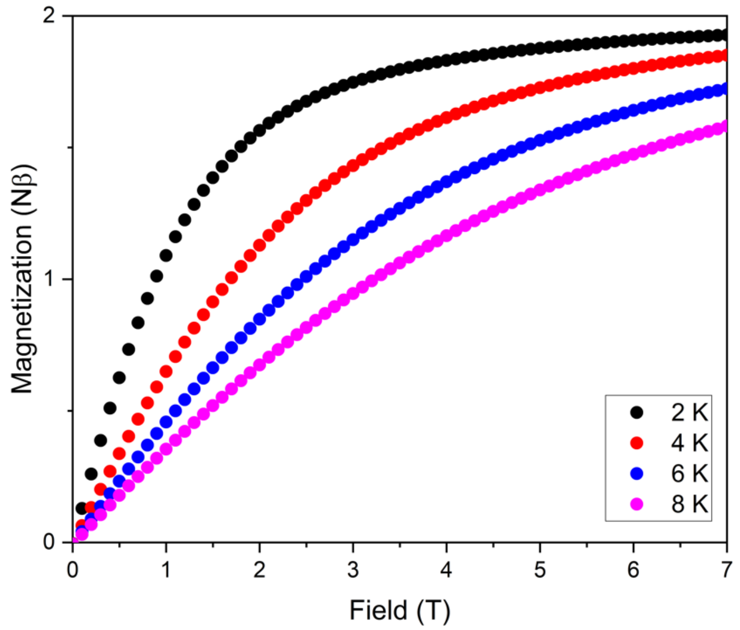

Figure S5. Variable field magnetization plots for **3-[Yb_2]$^{5+}$**.

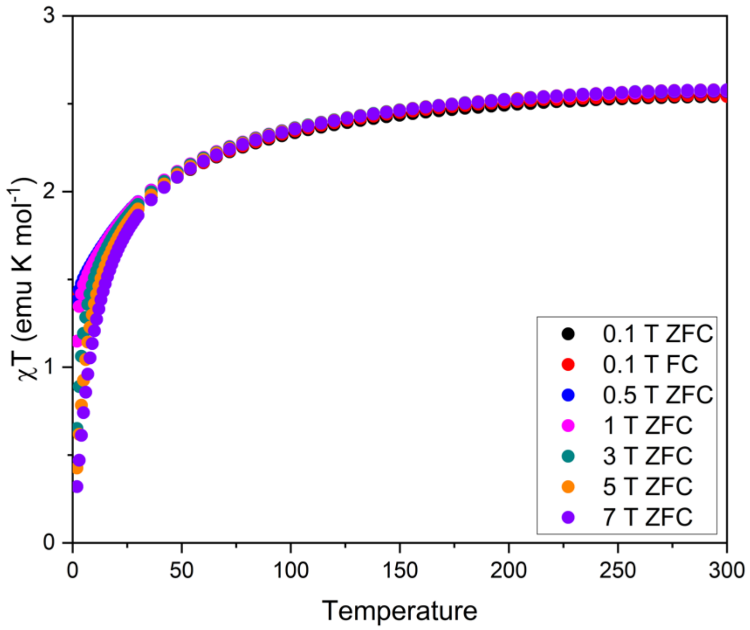

Figure S6. Variable field χT vs T plots for **5-[Yb]$^{3+}$**. The typically observed room temperature χT for one diamagnetic Yb^{2+} ion and one Yb^{3+} ion is 2.5-2.6 emu K mol^{-1}.

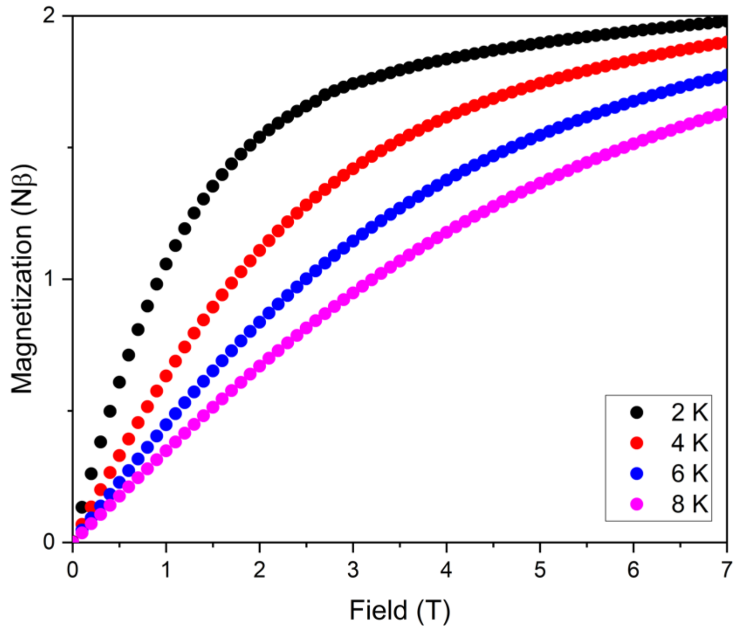

Figure S7. Variable field magnetization plots for **5-[Yb]$^{3+}$**.

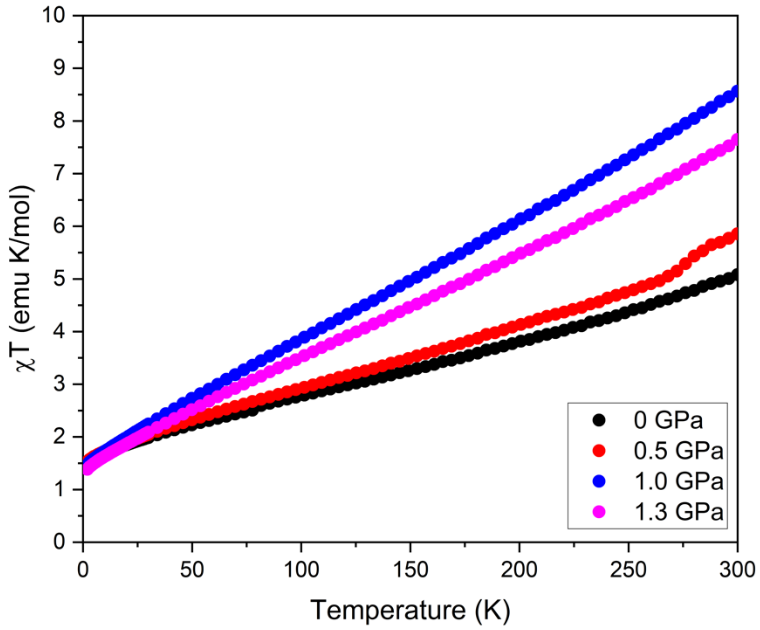

Figure S8. Variable pressure χT vs T plots for **3-[Yb$_2$]$^{5+}$** at 0.1 T applied field. The data above 50 K is dominated by temperature-independent magnetism (TIP). The TIP is present even with no applied pressure, suggesting it is an artifact of the pressure cell measurement.

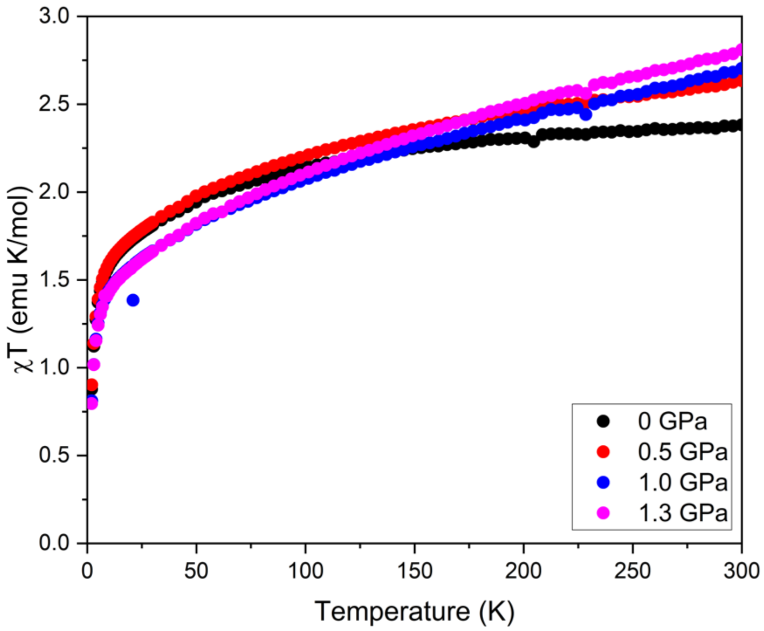

Figure S9. Variable pressure χT vs T plots for **3-[Yb$_2$]$^{5+}$** at 2.0 T applied field. The TIP present at lower field is suppressed, restoring the room temperature χT value of ~2.5 emu K/mol.

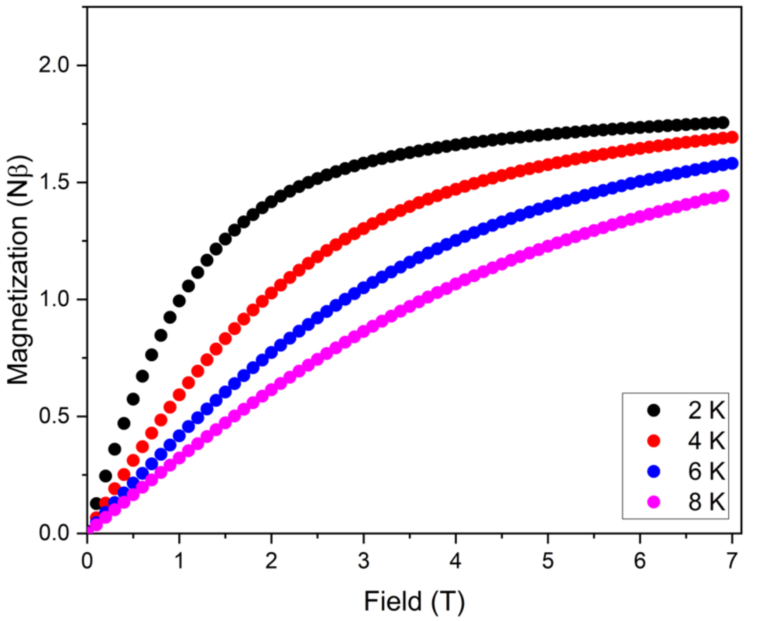

Figure S10. Variable field magnetization plots for **3-[Yb$_2$]$^{5+}$** at 1.3 GPa applied pressure. The saturation values are slightly lower than the unpressurized data in Figure S5, suggesting a small perturbation to the magnetic properties of **3-[Yb$_2$]$^{5+}$**.

Yb L_3-edge X-ray Absorption Near Edge Spectroscopy (XANES)

Samples were prepared in an argon glovebox at Stanford Synchrotron Radiation Lightsource (SSRL) due to the highly air-sensitive nature of these compounds. A mixture of the analyte and boron nitride (BN) was weighed, such that the edge jump for the absorbing atom was calculated to be at one absorption length in transmission (~8–12 mg of sample). The samples were diluted with BN (~15 mg), which had been dried at elevated temperature (250 °C) under vacuum (1×10^{-3} Torr) for 24 h prior to use. Samples were ground with a mortar and pestle.

Solid-state sample holders for the Yb samples consisted of an aluminum plate with a 3×15 mm^2 oval window and screw holes. One side of the plate was covered with 0.5 mm Kapton tape, and the sample was evenly loaded in the window. The powder was then secured by covering the sample with a second piece of 0.5 mm Kapton tape. A second layer of compound was painted onto a third piece of Kapton tape, which was subsequently fixed to the backside of the sample holder. The sample holder was then loaded onto a sample rod, taken out of the glovebox, and transported to the beamline while submerged within a liquid nitrogen cooling bath. Once at the beam, the rod with the sample was placed at 45° inside the Oxford $He_{(liq)}$ cryostat, which was precooled at 85 K and attached to the SSRL Beamline 11-2 rail. When the cryostat was closed, the system was put under vacuum and flushed with helium five times. The valve was closed, and the measurements were performed in the cryostat at 10 K.

The solid-state ytterbium complexes were characterized by Yb L_3-edge X-ray measurements. The X-ray absorption measurements were made at SSRL, under dedicated operating conditions (3.0 GeV, 5%, 500 mA using continuous top-off injections) on end station 11-2. With the use of a liquid-nitrogen-cooled double-crystal Si(220) (φ = 90) monochromator that employed collimating and focusing mirrors, a single energy was selected from the incident white beam. For Yb measurements, the beam was fully tuned at 8944 eV, and harmonic rejection was achieved with an Rh-coated mirror. The horizontal slit sizes were 10 mm, and vertical slit sizes were 1 mm in all measurements.

The cryostat was attached to the beamline 11-2 XAS rail (SSRL), which was equipped with three ionization chambers, through which nitrogen gas was continually flowed. One chamber was positioned before the helium beam pass and the cryostat (10 cm) to monitor the incident radiation (I_0). The second chamber was positioned after the cryostat (30 cm) so that sample transmission (I_1) could be evaluated against I_0 and so that the absorption coefficient (μ) could be calculated as $\ln(I_0/I_1)$. The third chamber (I_2; 30 cm) was positioned downstream from I_1 so that the XANES of a calibration foil could be measured against I_1. A potential of 1100 V was applied in series to the ionization chambers. A PIPS detector under argon was placed on one side of the cryostat (4 cm) to detect the fluorescence from the samples. The Yb samples were calibrated in situ to the energy of the first inflection point of the K-edge of a copper foil (8979 eV). Data were acquired in triplicate and averaged, aside from the data for **3-[Yb$_2$]$^{5+}$** which was observed degrading across 5 sequential scans (Figure S12). Background subtraction and normalization were performed in Athena.[12] The normalized data were fit using python code with a pseudo-voigt lineshape for each absorption feature and a logistic curve step function at the edge. Inflection points were determined by finding the energies for which the numerical second derivative was zero. An overlay of the experimental data for **1-[Yb$_2$]$^{6+}$**, **2-[Yb$_2$]$^{6+}$**, **3-[Yb$_2$]$^{5+}$**, and **5-[Yb]$^{3+}$** is given in figure S13.

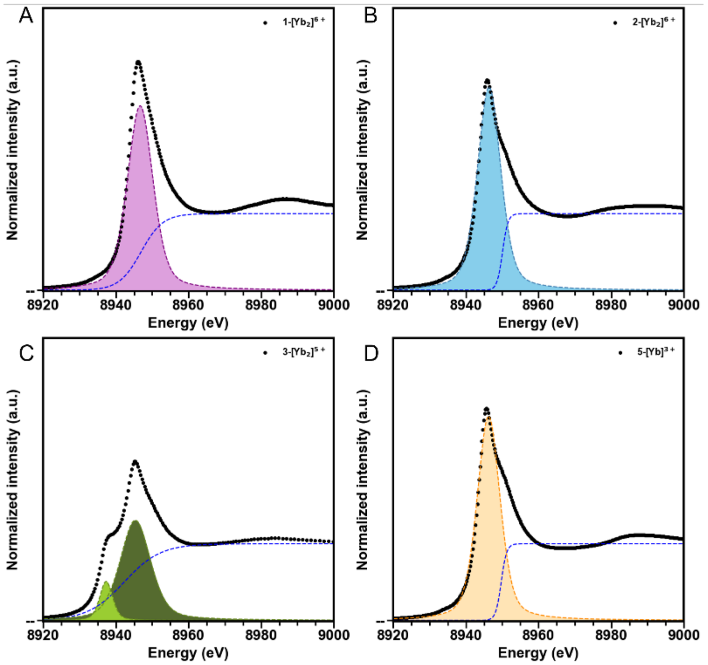

Figure S11. XANES fits for **1-[Yb₂]$^{6+}$** (A); **2-[Yb₂]$^{6+}$** (B); **3-[Yb₂]$^{5+}$** (C); and **5-[Yb]$^{3+}$** (D). Fitted peak energies are 8946.70(7) eV for **1-[Yb₂]$^{6+}$**, 8946.40(4) eV for **2-[Yb₂]$^{6+}$**, 8937.34(8) and 8945.37(7) eV for **3-[Yb₂]$^{5+}$**, and 8946.27(5) eV for **5-[Yb]$^{3+}$**.

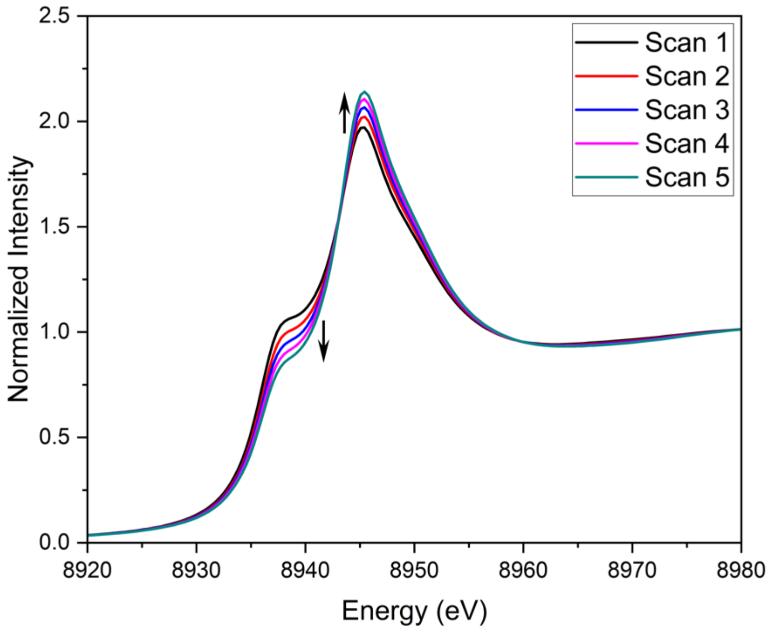

Figure S12. XANES spectra for **3-[Yb₂]$^{5+}$** showing loss of the lower energy Yb^{2+} signal and growth of the higher energy Yb^{3+} signal across sequential scans.

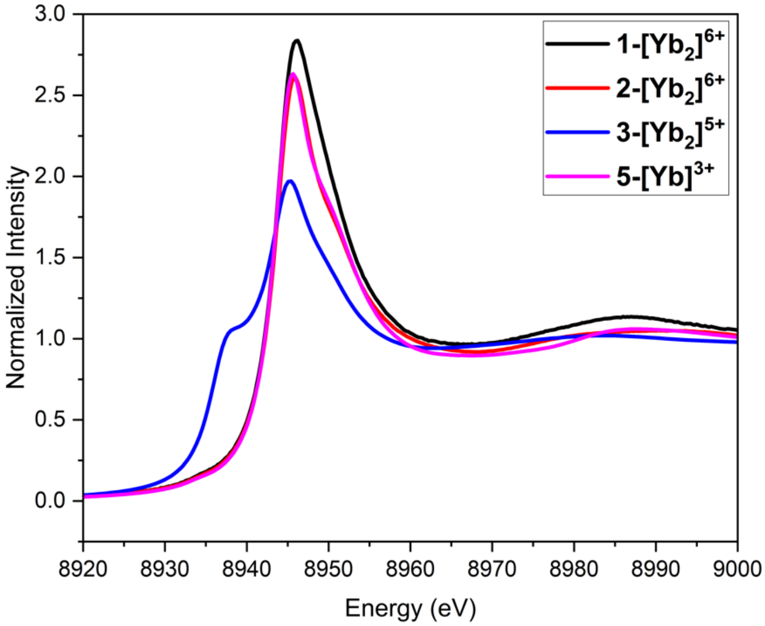

Figure S13. Overlay of the experimental XANES spectra for **1-[Yb$_2$]$^{6+}$**, **2-[Yb$_2$]$^{6+}$**, **3-[Yb$_2$]$^{5+}$**, and **5-[Yb]$^{3+}$**.

Quantum Chemical Calculations

All calculations employed the SARC2-QZVP (Yb) / def2-TZVP (P,N) / def2-SVP (C,H) basis set combination.[13, 14] Single point and TD-DFT calculations were performed on the unoptimized crystal structures using the CAM-B3LYP functional.[15] In all DFT calculations, relativistic effects were taken into account using the ZORA procedure.[16] To facilitate complete active space self-consistent field (CASSCF) calculations we have created truncated model geometries in which the R groups of the molecule were replaced by hydrogens. The positions of the hydrogen atoms were then optimized using the functional/basis set combination above except with the SARC-TZVP basis set on Yb and the BP86 functional.[17, 18]

To accurately calculate the *f*-*f* transitions and magnetic properties we have performed state averaged- CASSCF (SA-CASSCF) calculations. In the SA-CASSCF calculations, scalar relativistic effects were accounted for using the second-order Douglas-Kroll-Hess (DKH) procedure.[19] To account for dynamic correlation, the converged wave functions were subjected to N-electron valence perturbation theory to second order (NEVPT2).[20] In these calculations, spin-orbit coupling was accounted for by quasi-degenerate perturbation theory. For the Yb^{3+} complex as well as single site models employed in the Yb_2^{6+} and Yb_2^{5+} complexes, the active space consisted of the 13 *f*-electrons in seven *f*-orbitals averaged over all seven spin-free states. In the dimer complexes, the active space was extended to include all 14 f orbitals (seven from each metal site) and all corresponding *f*-electrons (26 for Yb_2^{6+} and 27 for Yb_2^{5+}). All calculations were performed using the Orca5.0 program package.[21, 22] Example input files are provided below.

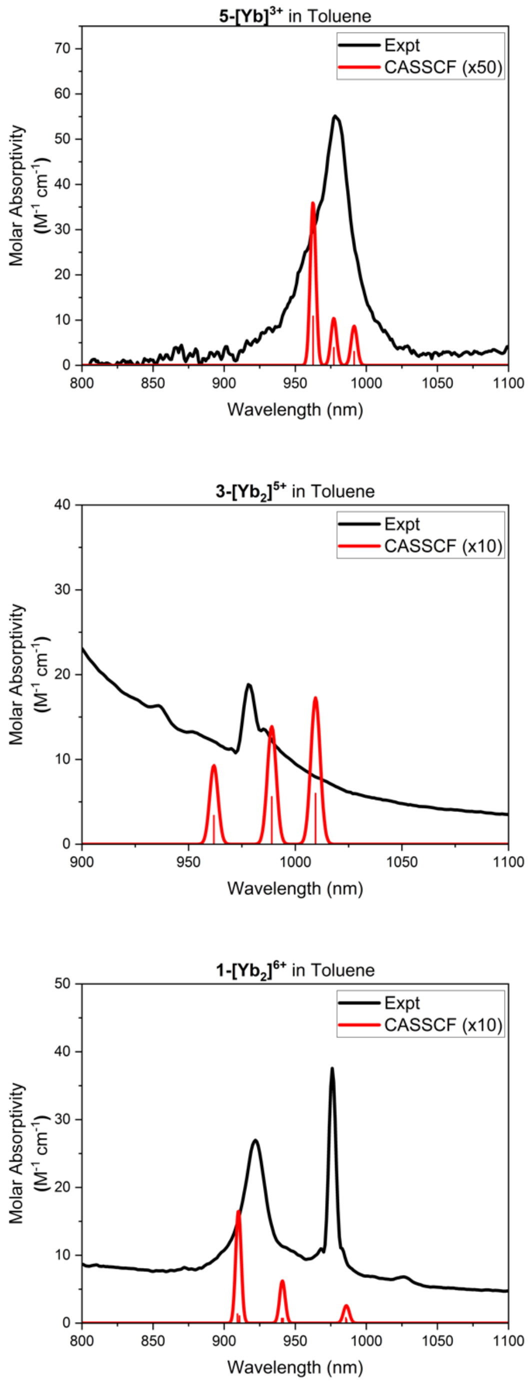

Figure S14. CASSCF predictions for *f*→*f* transitions in **1-[Yb$_2$]$^{6+}$**, **3-[Yb$_2$]$^{5+}$**, and **5-[Yb]$^{3+}$**.

Discussion of Metal-metal Distance Comparisons

In order to quantify the shortness of metal-metal bonds across a variety of transition metals, Cotton devised the formal shortness ratio (FSR), where the metal-metal distance is normalized by the sum of the covalent radii.[23, 24] For the covalent radii, Cotton selected the single bond radii, R(1), formulated by Pauling,[25] noting that the exact radii values are not so important as the consistency across the metals being compared. The Pauling R(1) radii work well for transition metals; however, when extending this methodology to lanthanide complexes, these radii are no longer ideal. In particular, the R(1) radii for europium and ytterbium are significantly larger than their neighbors. This is attributed to the derivation of R(1) from the metallic radii,[25] with europium and ytterbium having [Xe]$6s^2 4f^7$ and [Xe]$6s^2 4f^{14}$ configurations. A variety of other covalent radii have been published, [26-28]and the relative merits of any given method and tabulation are subject to debate.[29] By contrast, the Shannon ionic radii offer a consistent empirical trend, although they are significantly shorter than the tabulated covalent radii.[30] As not all oxidation states and coordination numbers are available, we use only the 6-coordinate trivalent radii in defining a new formal shortness ratio (FSR′) for use with multi-lanthanide complexes:

$$\mathrm{FSR}' = \frac{D_{A-B}}{2(R_A + R_B)}$$

Where $D_{A\text{-}B}$ is the internuclear distance and R_A and R_B are the 6-coordinate trivalent Shannon radii of atoms A and B. The coefficient of 2 in the denominator scales the value to be similar to conventionally defined FSR, ***though FSR′ and FSR values should not be directly compared to each other.***

Table S3 below shows a selection of homodilanthanide complexes with particularly short contacts (FSR < 1), as well as the recent Ln-Ln bonded complexes from Gould and coworkers. There are several important observations that can be made from these data. First, non-bonding short contacts are available across the +2, +3, and +4 oxidation states, depending on the lanthanide in question. Second, there is no rigorous correlation between internuclear distance and bonding between lanthanide ions. However, within a conserved ligand framework, such as the $Cp^{iPr5}{}_2Ln_2I_3$ complexes, the bonding gadolinium, terbium, and dysprosium complexes have significantly shorter internuclear distances than in the non-bonding thulium complex. This serves as a reminder that the context in which internuclear distances are compared is critical to shaping the conclusions that can be made from such comparisons.

Table S3. Representative selection of homodilanthanide complexes with short lanthanide-lanthanide distances.

Compound	Oxidation States	$D_{A\text{-}B}$	FSR′	Ref
$[(Cp^{Ar5})La(\mu\text{-}H)]_2$	2,2	3.4266(4)	0.830	[31]
$K_4[Ce(O_2)(EDTA)]_2\bullet 14H_2O$	4,4	3.1953(5)	0.791	[32]
$[(OEPG)Sm_2(Et_2O)_2]^a$	2,2	3.3159(5)	0.865	[33]
$[Tb_4L_2(NO_3)_4(MeOH)_2(\mu_4\text{-}O)]^b$	3,3	3.389(1)	0.918	[34]
$[Li(THF)_4][(C_5Me_4SiMe_3)_4Dy_4(\mu\text{-}Cl)(\mu\text{-}H)_8]$	3,3	3.310(2)	0.907	[35]
$[(pdl')(pdl'^{-1H})(pdl'^{-2H})M_2(thf)_2]^c$	3,3	3.1372(2)	0.881	[36]
$[(Me_4TACD)_2Lu_2(\mu\text{-}H)_4][BAr^F\text{-}24]_2^d$	3,3	2.9270(6)	0.850	[37]
$[(Cp^{iPr5})_2Gd_2I_3]$	$2,3^e$	3.769(1)	1.00	[38]
$[(Cp^{iPr5})_2Tb_2I_3]$	$2,3^e$	3.732(1)	1.01	[38]
$[(Cp^{iPr5})_2Dy_2I_3]$	$2,3^e$	3.713(1)	1.02	[38]
$[(Cp^{iPr5})_2Tm_2I_3]$	2,3	3.960(1)	1.13	[38]
**1-$[Yb_2]^{6+}$**	3,3	3.4133(8)	0.983	This work
**2-$[Yb_2]^{6+}$**	3,3	3.3679(6)	0.970	This work
**3-$[Yb_2]^{5+}$**	2,3	2.9507(8)	0.850	This work
**4-$[Yb_2]^{5+}$**	2,3	3.38844(8)	0.976	This work

^{a}OEPC = octaethylporphyrinogen

$^b H_3L$ = 1,3-bis(2-hydroxy-3-methoxybenzylamino)propan-2-ol.

^{c}pdl′ = 2,4,-$^tBu_2C_5H_5$ anion, with $^{-1H}$ and $^{-2H}$ representing subsequent deprotonations to di- and tri-anions.

$^d Me_4TACD$ = 1,4,7,10-tetramethyl-1,4,7,10-tetraazacyclododecane.

^{e}These systems are more accurately described as 2.5,2.5 due to the delocalized σ bonding orbital.

NMR Spectra

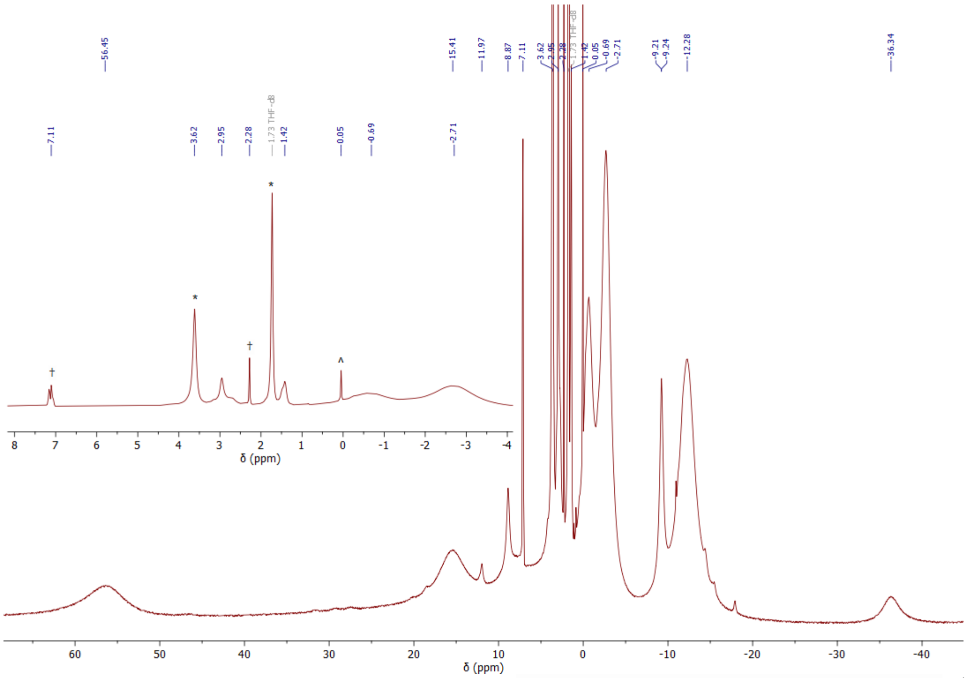

Figure S15. ^{1}H NMR of **1-[Yb$_2$]$^{6+}$** in THF-d$_8$ with 5 Hz exponential line broadening. * = THF-d$_8$, $^{\wedge}$ = grease, † = toluene.

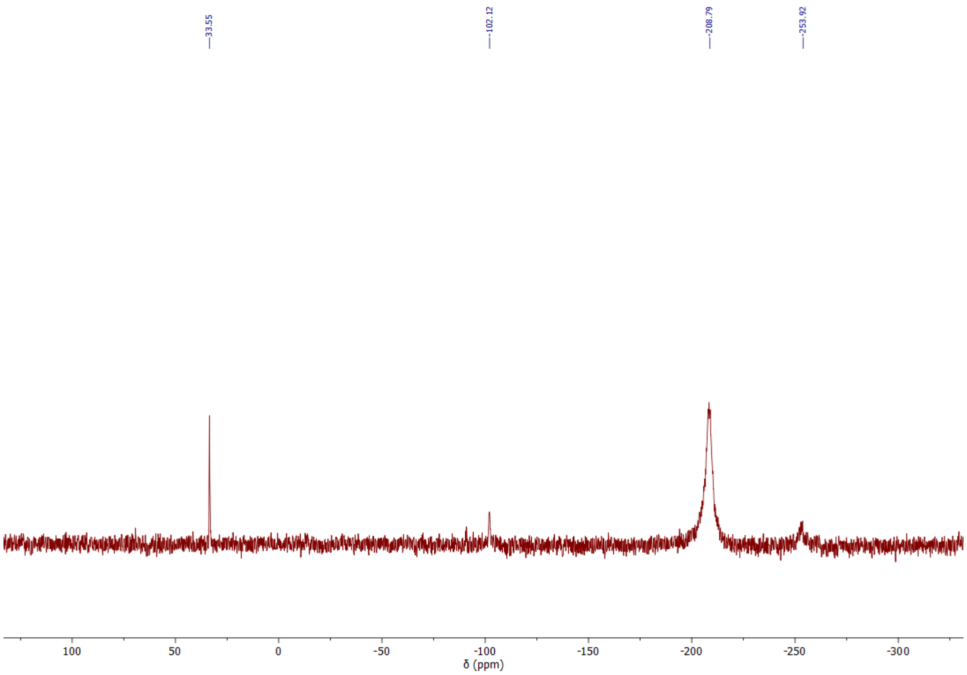

Figure S16. ^{31}P{^{1}H} NMR of **1-[Yb$_2$]$^{6+}$** in THF-d$_8$ with 10 Hz exponential line broadening.

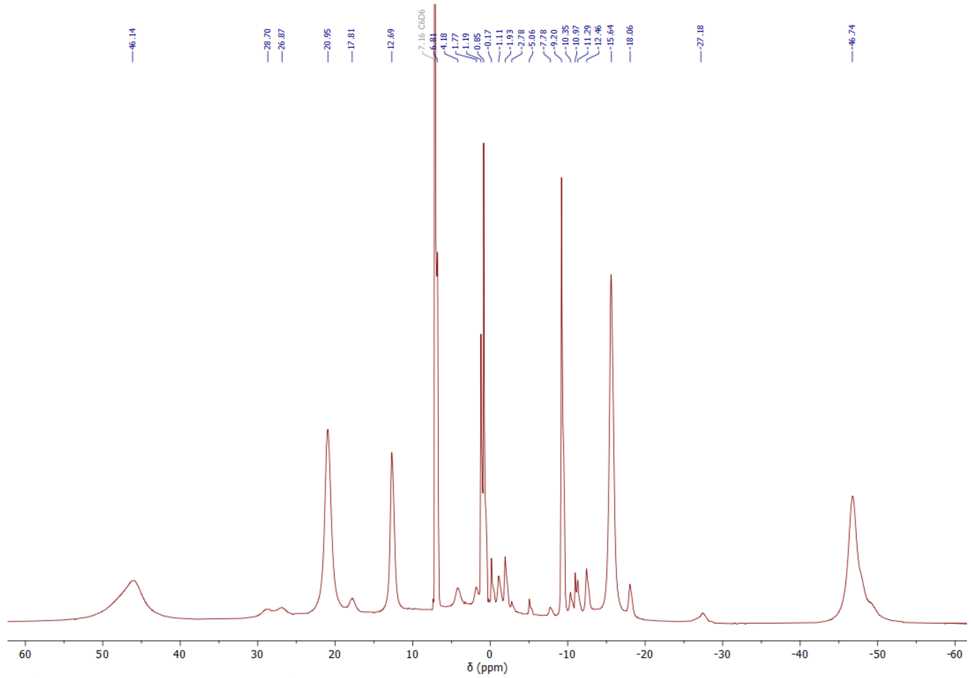

Figure S17. ^{1}H NMR of **2-[Yb$_2$]$^{6+}$** in C_6D_6 with 5 Hz exponential line broadening.

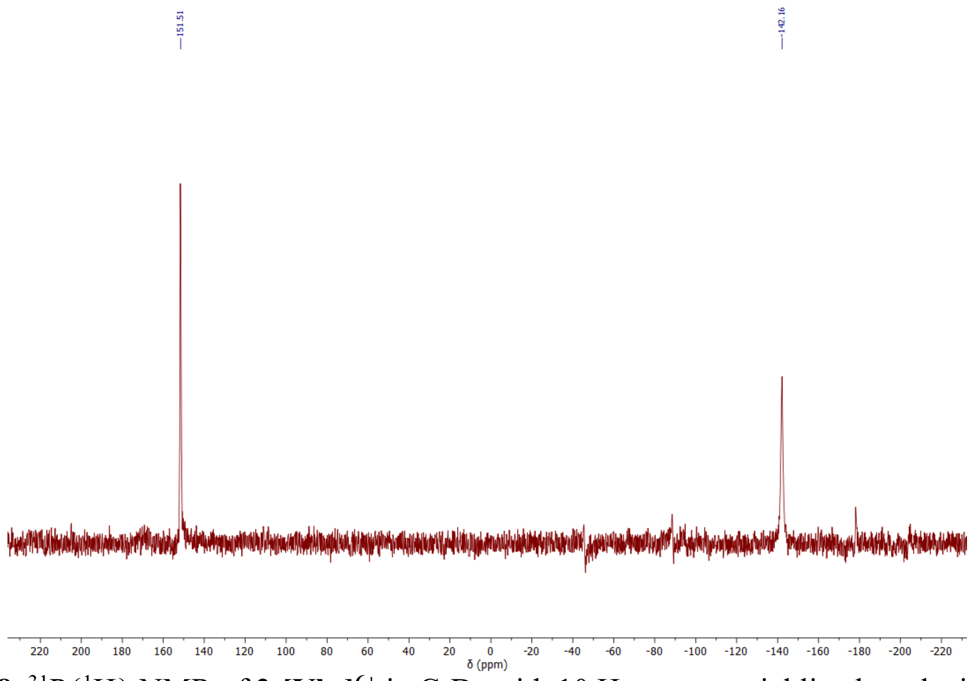

Figure S18. $^{31}P\{^{1}H\}$ NMR of **2-[Yb$_2$]$^{6+}$** in C_6D_6 with 10 Hz exponential line broadening.

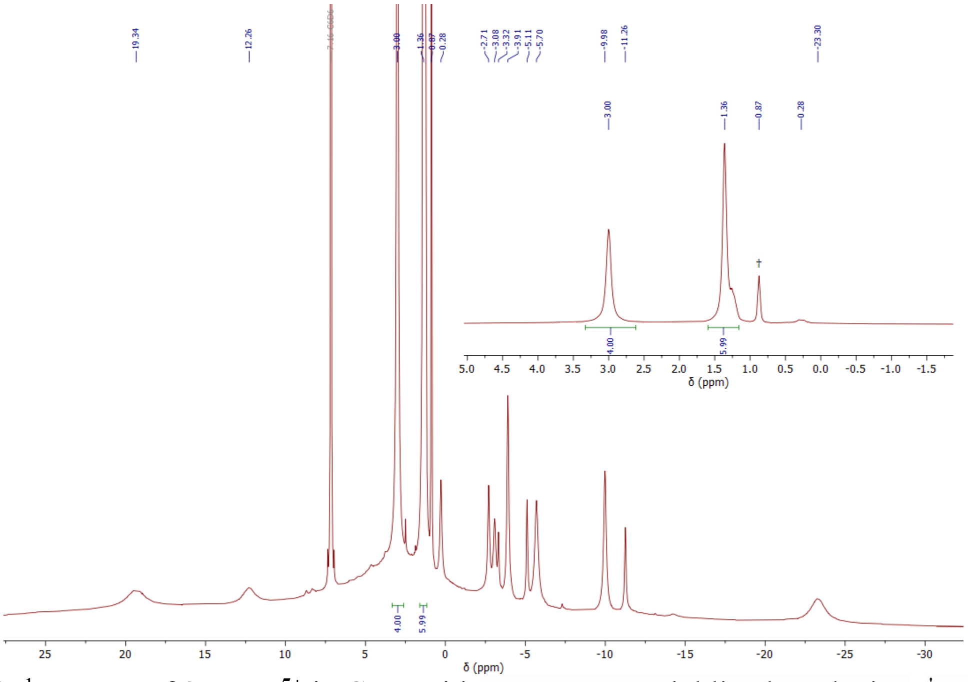

Figure S19. ^{1}H NMR of **3-[Yb$_2$]$^{5+}$** in C_6D_6 with 5 Hz exponential line broadening. † = pentane.

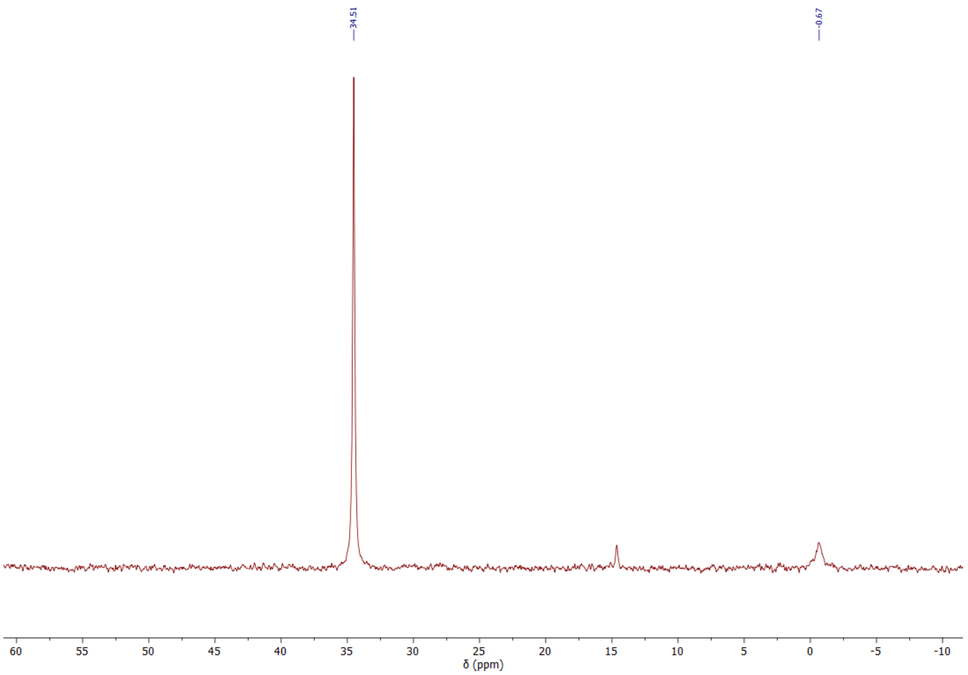

Figure S20. ^{31}P{^{1}H} NMR of **3-[Yb$_2$]$^{5+}$** in C_6D_6 with 10 Hz exponential line broadening.

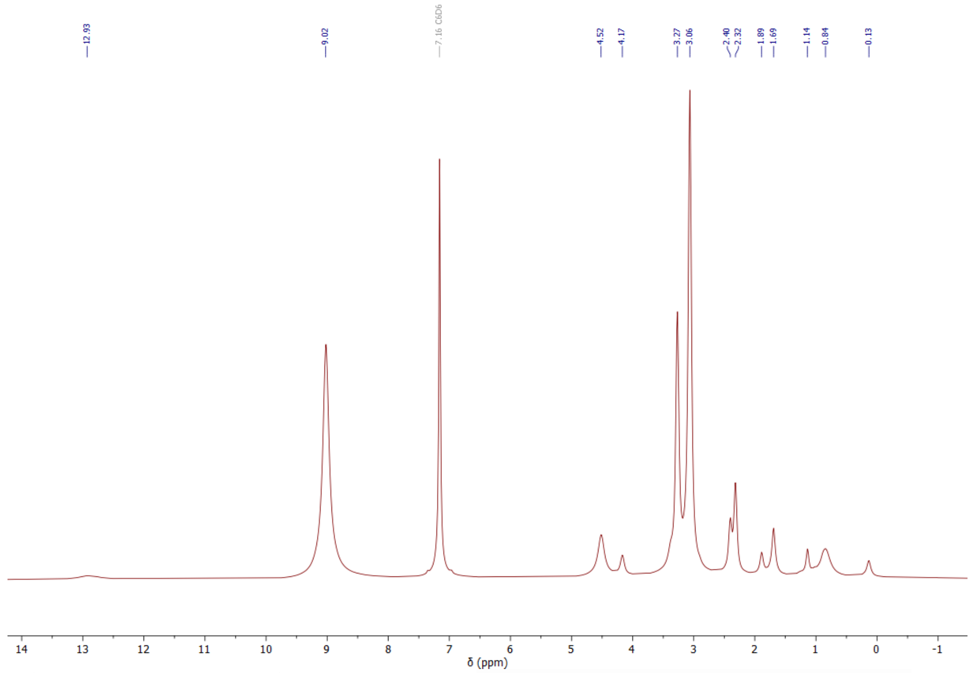

Figure S21. ^{1}H NMR of **5-[Yb]$^{3+}$** in C_6D_6 with 5 Hz exponential line broadening.

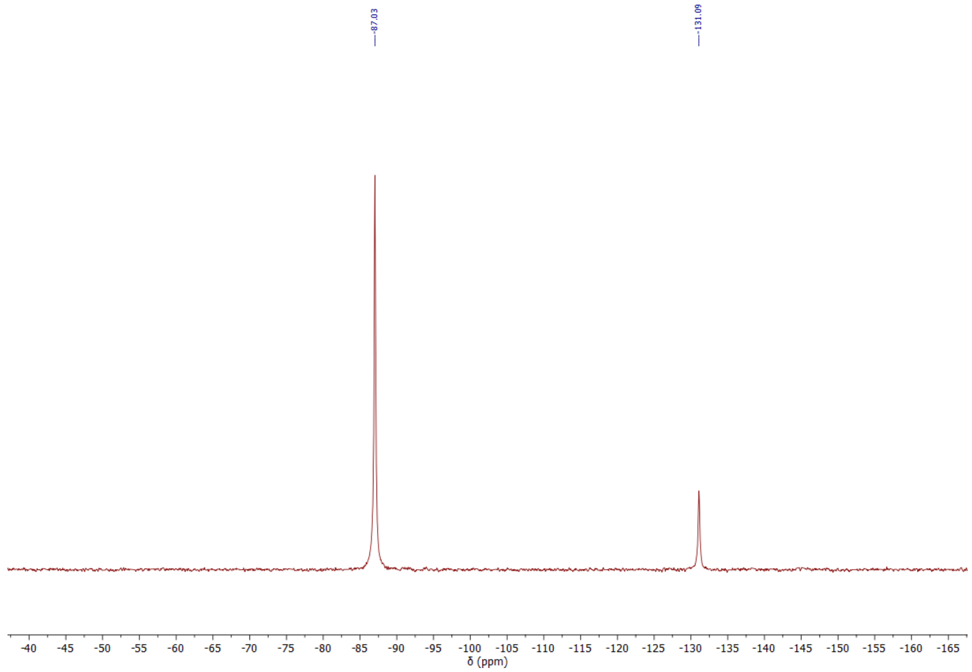

Figure S22. $^{31}P\{^{1}H\}$ NMR of **5-[Yb]$^{3+}$** in C_6D_6 with 10 Hz exponential line broadening.

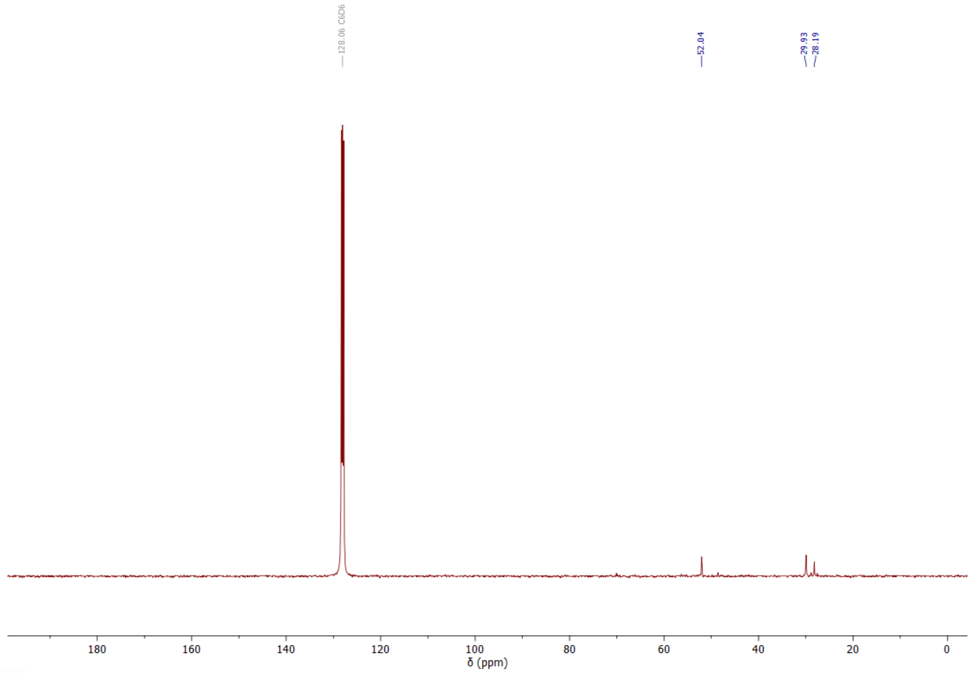

Figure S23. $^{13}C\{^1H\}$ NMR of **5-[Yb]$^{3+}$** in C_6D_6 with 5 Hz exponential line broadening.

UV/vis Spectral Fits

Spectral fits in the UV/vis region of the electronic absorption spectra of **3-[Yb_2]$^{5+}$** were performed using the curve fitting package in MatLab. Gaussian functions were used to approximate the two features between 400 nm and 900 nm. A third function was used to approximate the high energy feature centered at 379 nm. The molar absorptivity of this third peak was initially set to 700 M^{-1} cm^{-1} based on the spectrum of a diluted sample. Fits were conducted in units of molar absorptivity (M^{-1} cm^{-1}) and energy (cm^{-1}). A table of the optimized Gaussian parameters across solvents and temperatures, Table S4, is provided below.

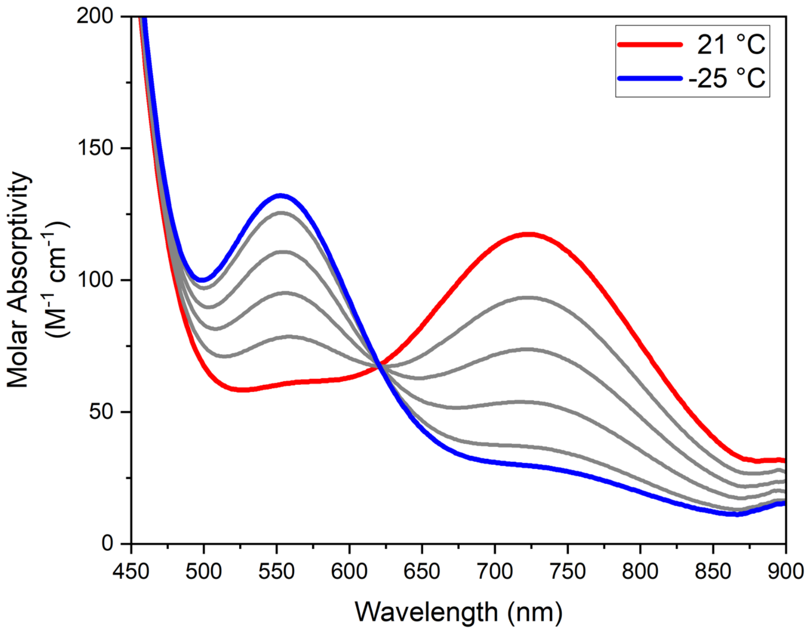

Figure S24. Variable temperature electronic absorption spectra for **3-[Yb_2]$^{5+}$** in Et_2O between 21 and -25 °C, with an isosbestic point at 621 nm demonstrating direct conversion between the closed and open (solvated) configurations. Gray lines represent data taken at ~9 ° increments (9, 0, -9, and -18 °C).

Diethyl Ether

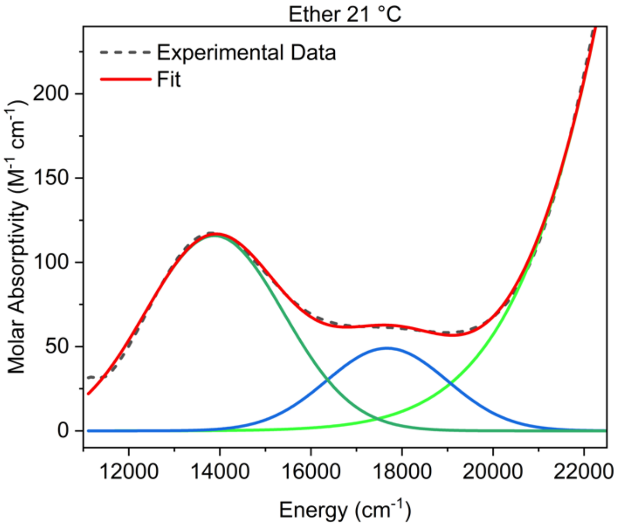

Figure S25. Gaussian fit for **3-[Yb_2]$^{5+}$** in diethyl ether at 21 °C. The blue, dark green, and lime green lines represent the individual Gaussian peaks that are added together in the overall fit (red line).

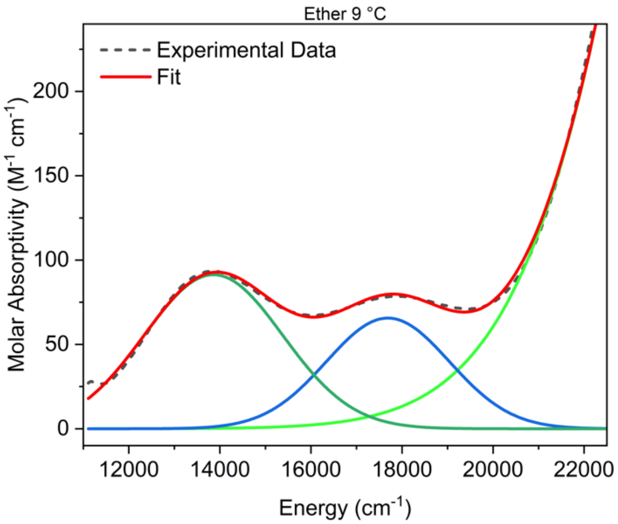

Figure S26. Gaussian fit for **3-[Yb_2]$^{5+}$** in diethyl ether at 9 °C. The blue, dark green, and lime green lines represent the individual Gaussian peaks that are added together in the overall fit (red line).

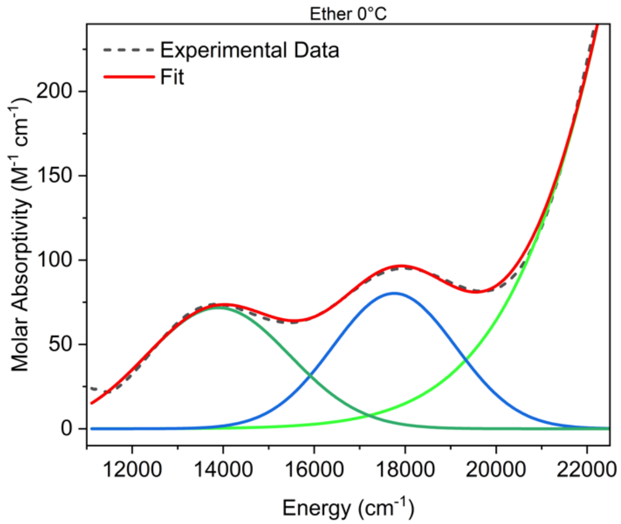

Figure S27. Gaussian fit for **3-[Yb$_2$]$^{5+}$** in diethyl ether at 0 °C. The blue, dark green, and lime green lines represent the individual Gaussian peaks that are added together in the overall fit (red line).

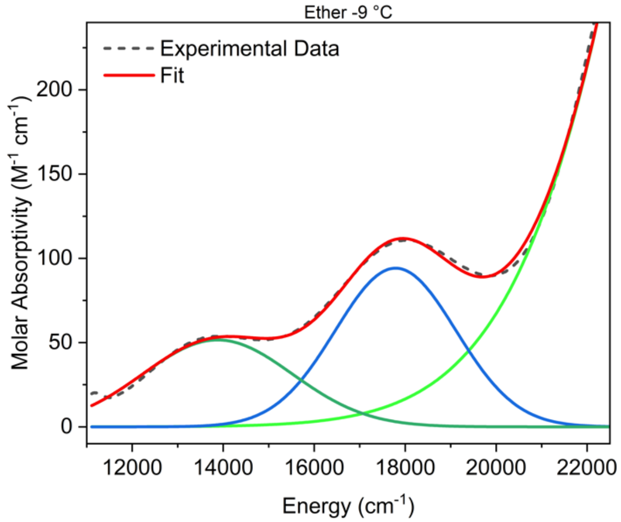

Figure S28. Gaussian fit for **3-[Yb$_2$]$^{5+}$** in diethyl ether at -9 °C. The blue, dark green, and lime green lines represent the individual Gaussian peaks that are added together in the overall fit (red line).

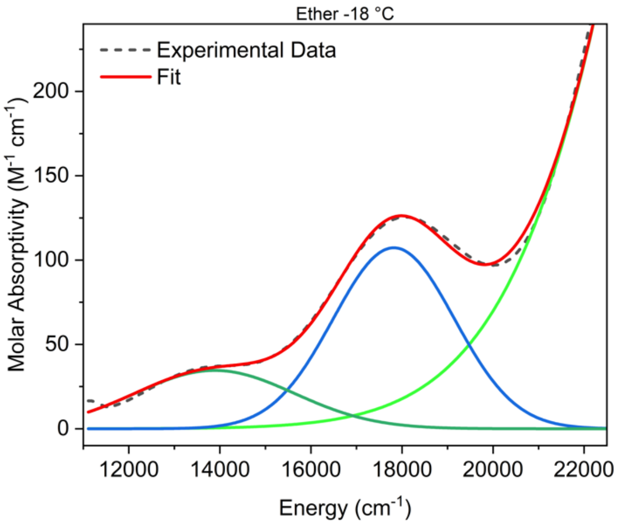

Figure S29. Gaussian fit for **3-[Yb$_2$]$^{5+}$** in diethyl ether at -18 °C. The blue, dark green, and lime green lines represent the individual Gaussian peaks that are added together in the overall fit (red line).

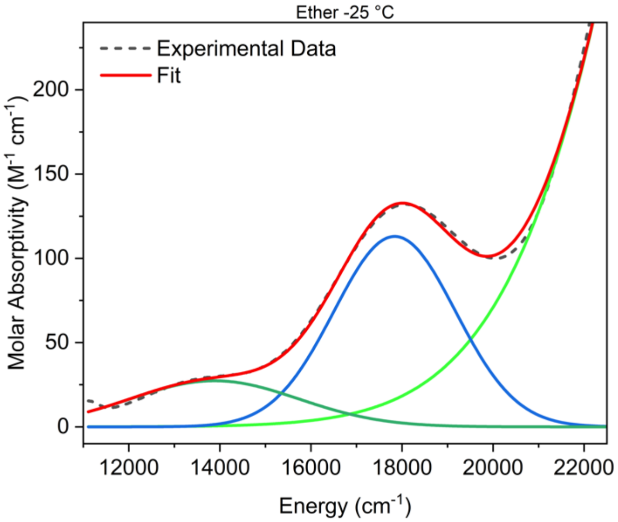

Figure S30. Gaussian fit for **3-[Yb$_2$]$^{5+}$** in diethyl ether at -25 °C. The blue, dark green, and lime green lines represent the individual Gaussian peaks that are added together in the overall fit (red line).

Hexanes

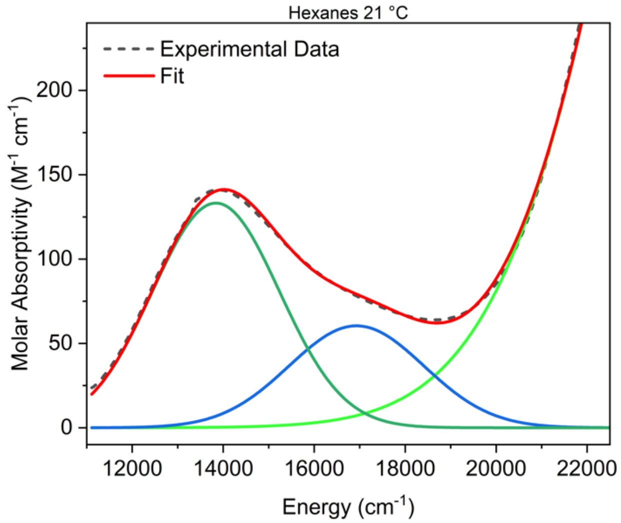

Figure S31. Gaussian fit for **3-[Yb$_2$]$^{5+}$** in hexanes at 21 °C. The blue, dark green, and lime green lines represent the individual Gaussian peaks that are added together in the overall fit (red line).

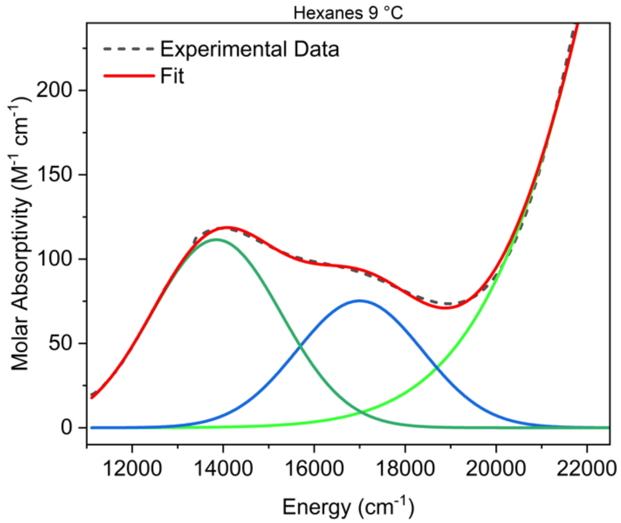

Figure S32. Gaussian fit for **3-[Yb$_2$]$^{5+}$** in hexanes at 9 °C. The blue, dark green, and lime green lines represent the individual Gaussian peaks that are added together in the overall fit (red line).

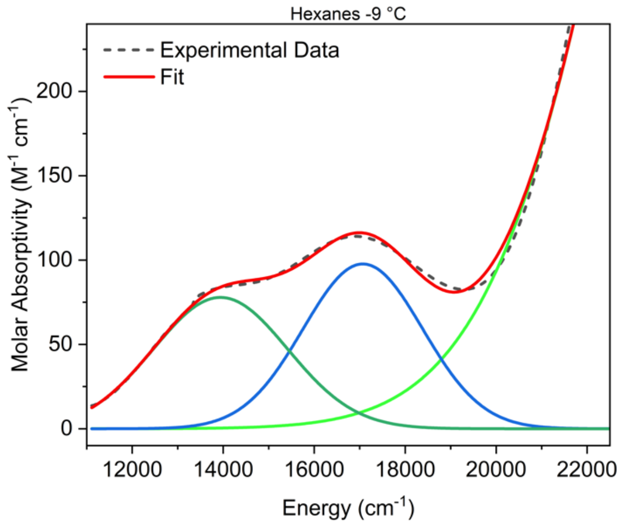

Figure S33. Gaussian fit for **3-[Yb$_2$]$^{5+}$** in hexanes at -9 °C. The blue, dark green, and lime green lines represent the individual Gaussian peaks that are added together in the overall fit (red line).

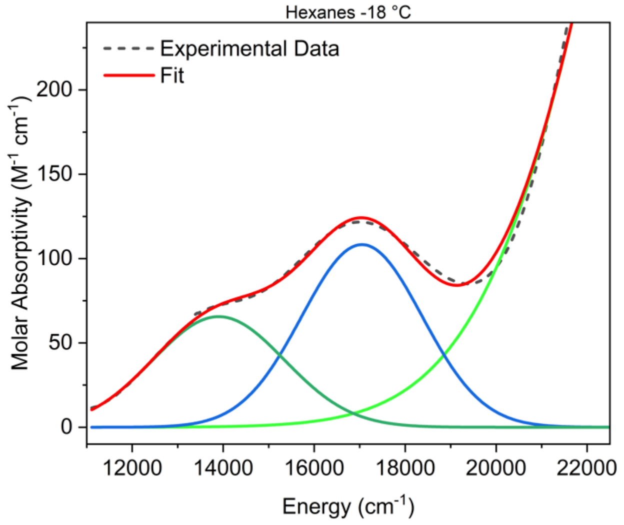

Figure S34. Gaussian fit for **3-[Yb$_2$]$^{5+}$** in hexanes at -18 °C. The blue, dark green, and lime green lines represent the individual Gaussian peaks that are added together in the overall fit (red line).

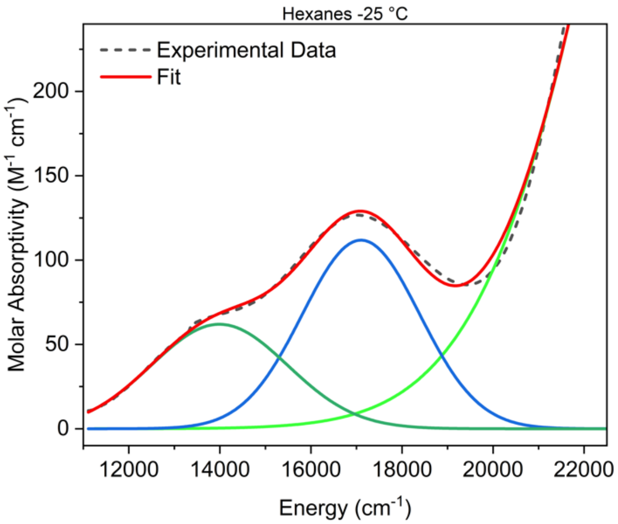

Figure S35. Gaussian fit for **3-[Yb$_2$]$^{5+}$** in hexanes at -25 °C. The blue, dark green, and lime green lines represent the individual Gaussian peaks that are added together in the overall fit (red line).

Toluene

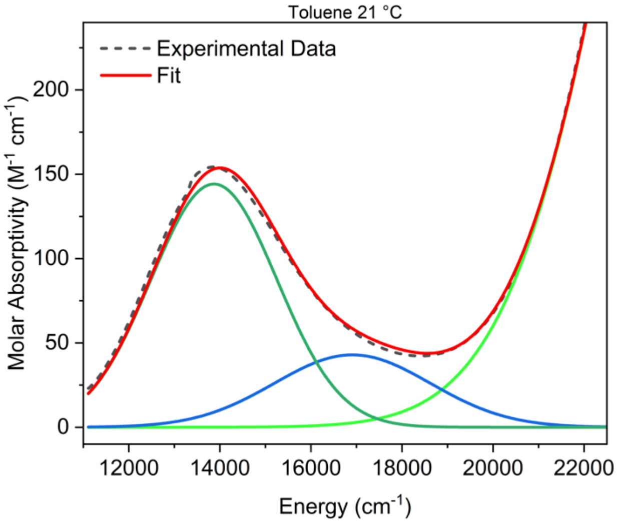

Figure S36. Gaussian fit for **3-[Yb$_2$]$^{5+}$** in toluene at 21 °C. The blue, dark green, and lime green lines represent the individual Gaussian peaks that are added together in the overall fit (red line).

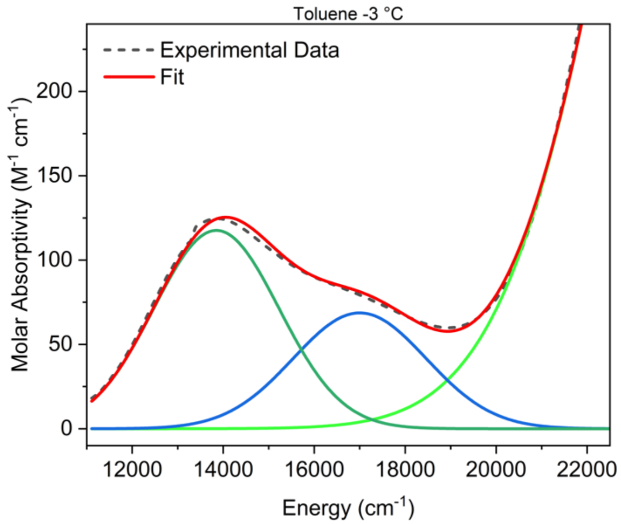

Figure S37. Gaussian fit for **3-[Yb$_2$]$^{5+}$** in toluene at -3 °C. The blue, dark green, and lime green lines represent the individual Gaussian peaks that are added together in the overall fit (red line).

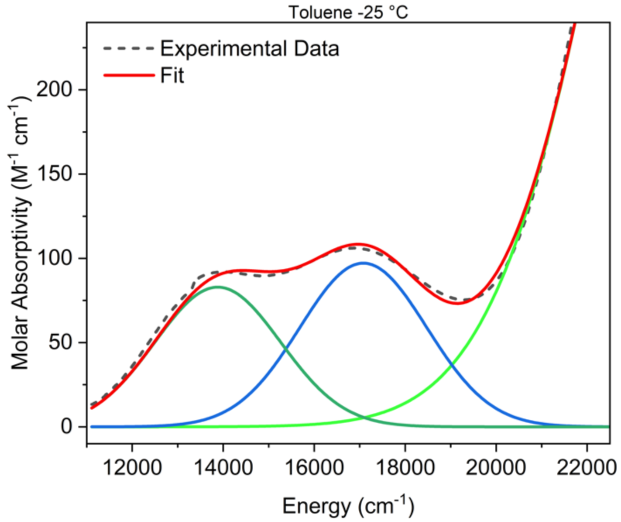

Figure S38. Gaussian fit for **3-[Yb$_2$]$^{5+}$** in toluene at -25 °C. The blue, dark green, and lime green lines represent the individual Gaussian peaks that are added together in the overall fit (red line).

Table S4. Fit parameters for solvent and temperature dependent electronic absorption spectra of **3-[Yb$_2$]$^{5+}$**.

		**Open configuration**				**Closed configuration**			
Solvent	Temp (°C)	ε (M^{-1} cm^{-1})	ν_{max} (cm^{-1})	$\Delta\nu_{1/2}$ (cm^{-1})	Γ	ε (M^{-1} cm^{-1})	ν_{max} (cm^{-1})	$\Delta\nu_{1/2}$ (cm^{-1})	Γ
Ether	21	49.0	17670	3129	0.51	115.8	13879	3575	0.34
	9	65.6	17690	3184	0.49	91.3	13859	3585	0.34
	0	80.3	17760	3120	0.49	71.7	13889	3712	0.31
	-9	94.2	17790	3127	0.48	51.6	13877	3878	0.28
	-18	107.3	17820	3140	0.47	34.6	13885	4124	0.24
	-25	113.0	17840	3145	0.46	27.3	13881	4359	0.19
Hexanes	21	61.4	16930	3490	0.44	133.2	13840	3325	0.38
	9	75.2	17000	3265	0.46	111.5	13850	3364	0.38
	-9	97.7	17070	3092	0.48	77.9	13940	3480	0.36
	-18	108.3	17050	3120	0.46	65.6	13900	3418	0.37
	-25	111.8	17100	3037	0.47	61.9	13990	3533	0.35
Toluene	21	42.9	16910	4040	0.35	144.2	13870	3262	0.40
	-3	68.7	17000	3443	0.42	117.6	13850	3244	0.40
	-25	97.1	17080	3287	0.42	84.9	13880	3255	0.40

Crystallographic Information

1-[Yb_2]$^{6+}$

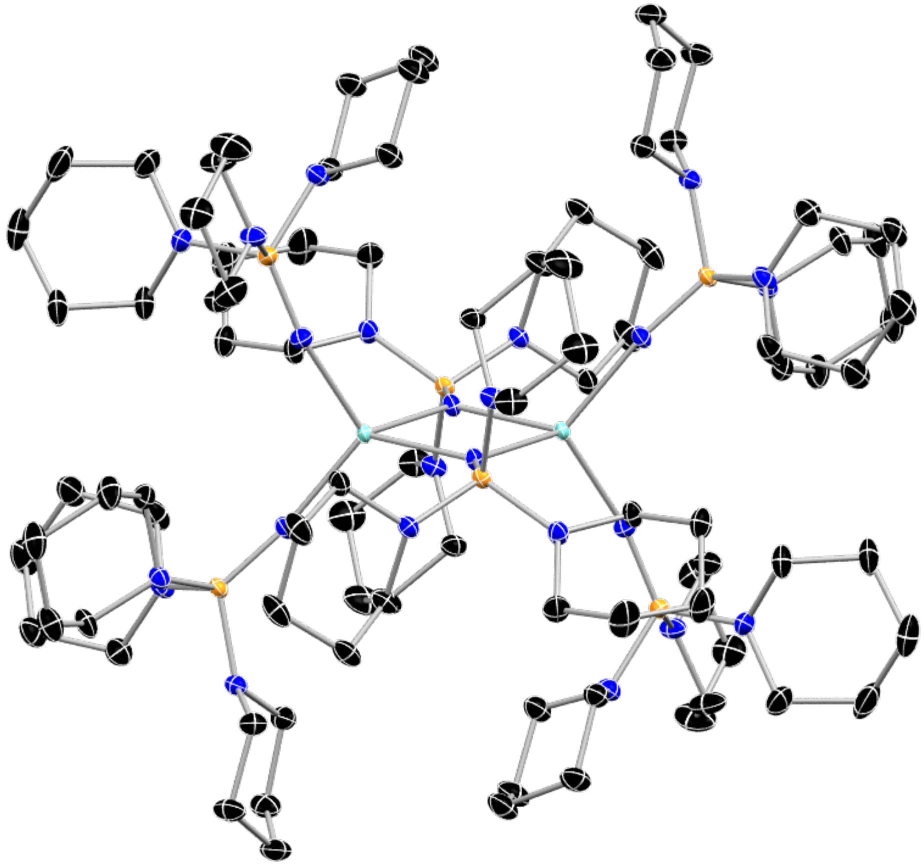

Crystal data and structure refinement for 1-$[Yb_2]^{6+}$.

Identification code	**1-$[Yb_2]^{6+}$**
Empirical formula	$C_{102}H_{204}N_{24}O_3P_6Yb_2$
Formula weight	2346.78
Temperature/K	102(1)
Crystal system	monoclinic
Space group	$P2_1/n$
a/Å	15.7562(2)
b/Å	16.2258(2)
c/Å	22.5641(4)
α/°	90
β/°	90.1568(16)
γ/°	90
Volume/Å^3	5768.65(16)
Z	2
ρ_{calc}g/cm^3	1.351
μ/mm^{-1}	1.752
F(000)	2476.0
Crystal size/mm^3	0.38 × 0.31 × 0.19
Radiation	Mo Kα (λ = 0.71073)
2Θ range for data collection/°	3.092 to 74.07
Index ranges	$-26 \le h \le 26$, $-23 \le k \le 27$, $-38 \le l \le 38$
Reflections collected	82672
Independent reflections	28259 [R_{int} = 0.0763, R_{sigma} = 0.0657]
Data/restraints/parameters	28259/172/644
Goodness-of-fit on F^2	1.025
Final R indexes [I>=2σ (I)]	R_1 = 0.0465, wR_2 = 0.1269
Final R indexes [all data]	R_1 = 0.0549, wR_2 = 0.1321
Largest diff. peak/hole / e Å^{-3}	3.20/-1.84

2-[$Yb_2]^{6+}$

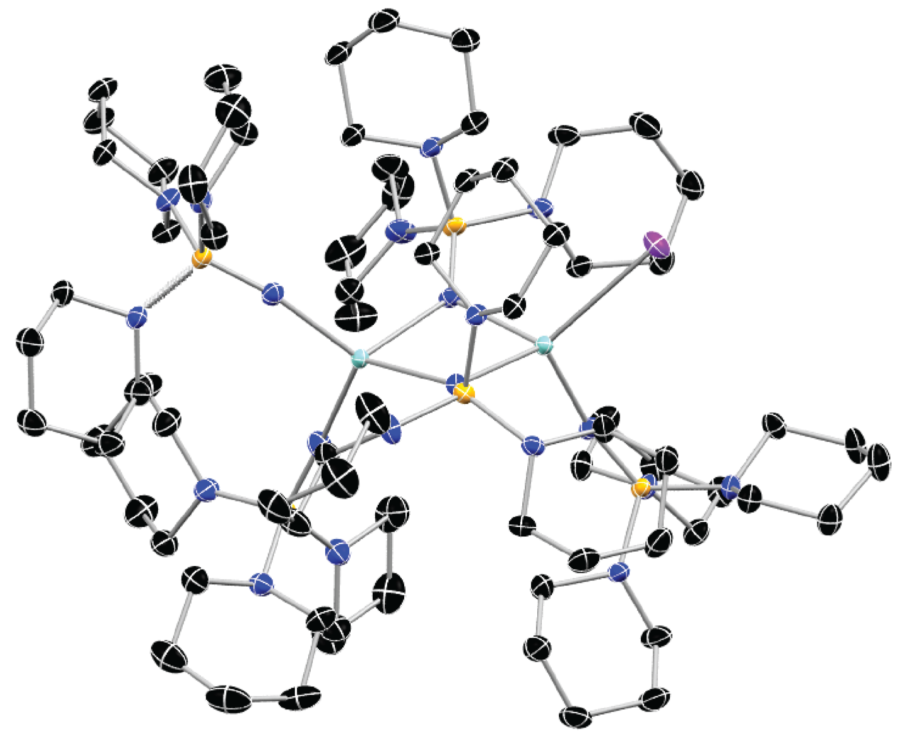

Crystal data and structure refinement for 2-[Yb_2]$^{6+}$.

Identification code	**2-[Yb_2]$^{6+}$**
Empirical formula	$C_{5.56}H_{11.11}I_{0.07}N_{1.48}P_{0.37}Yb_{0.15}$
Formula weight	145.18
Temperature/K	109.78
Crystal system	triclinic
Space group	P-1
a/Å	14.6571(8)
b/Å	14.7761(9)
c/Å	25.5541(15)
α/°	92.705(2)
β/°	97.818(2)
γ/°	116.804(2)
Volume/Å^3	4856.8(5)
Z	27
ρ_{calc}g/cm^3	1.340
μ/mm^{-1}	2.361
F(000)	2016.0
Crystal size/mm^3	0.471 × 0.316 × 0.232
Radiation	MoKα (λ = 0.71073)
2Θ range for data collection/°	4.716 to 66.262
Index ranges	$-22 \leq h \leq 22$, $-22 \leq k \leq 22$, $-39 \leq l \leq 38$
Reflections collected	93084
Independent reflections	36925 [R_{int} = 0.0345, R_{sigma} = 0.0391]
Data/restraints/parameters	36925/44/966
Goodness-of-fit on F^2	1.019
Final R indexes [I>=2σ (I)]	R_1 = 0.0262, wR_2 = 0.0644
Final R indexes [all data]	R_1 = 0.0315, wR_2 = 0.0670
Largest diff. peak/hole / e Å^{-3}	1.62/-0.82

3-[Yb$_2$]$^{5+}$

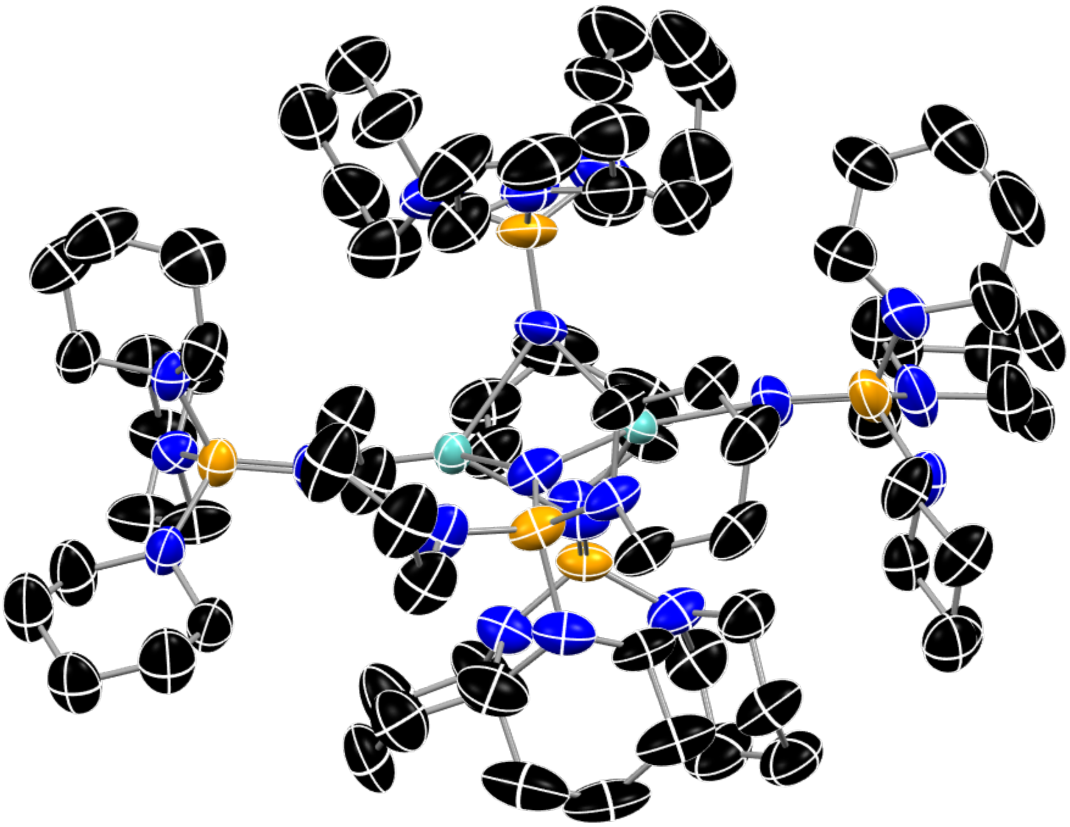

Crystal data and structure refinement for 3-$[Yb_2]^{5+}$.

Identification code	**3-$[Yb_2]^{5+}$**
Empirical formula	$C_{79}H_{160}N_{20}OP_5Yb_2$
Formula weight	1907.19
Temperature/K	100(2)
Crystal system	monoclinic
Space group	$P2_1$
a/Å	13.8770(9)
b/Å	27.7567(15)
c/Å	14.0035(9)
α/°	90
β/°	119.503(2)
γ/°	90
Volume/Å^3	4694.4(5)
Z	2
ρ_{calc}g/cm^3	1.349
μ/mm^{-1}	2.117
F(000)	1994.0
Crystal size/mm^3	0.219 × 0.191 × 0.147
Radiation	MoKα (λ = 0.71073)
2Θ range for data collection/°	4.448 to 52.8
Index ranges	$-17 \leq h \leq 17$, $-34 \leq k \leq 34$, $-17 \leq l \leq 17$
Reflections collected	77901
Independent reflections	19128 [R_{int} = 0.0865, R_{sigma} = 0.0704]
Data/restraints/parameters	19128/1862/ 977
Goodness-of-fit on F^2	1.065
Final R indexes [I>=2σ (I)]	R_1 = 0.0580, wR_2 = 0.1255
Final R indexes [all data]	R_1 = 0.0797, wR_2 = 0.1402
Largest diff. peak/hole / e Å^{-3}	2.54/-1.03
Flack parameter	-0.011(6)

4-[Yb$_2$]$^{5+}$

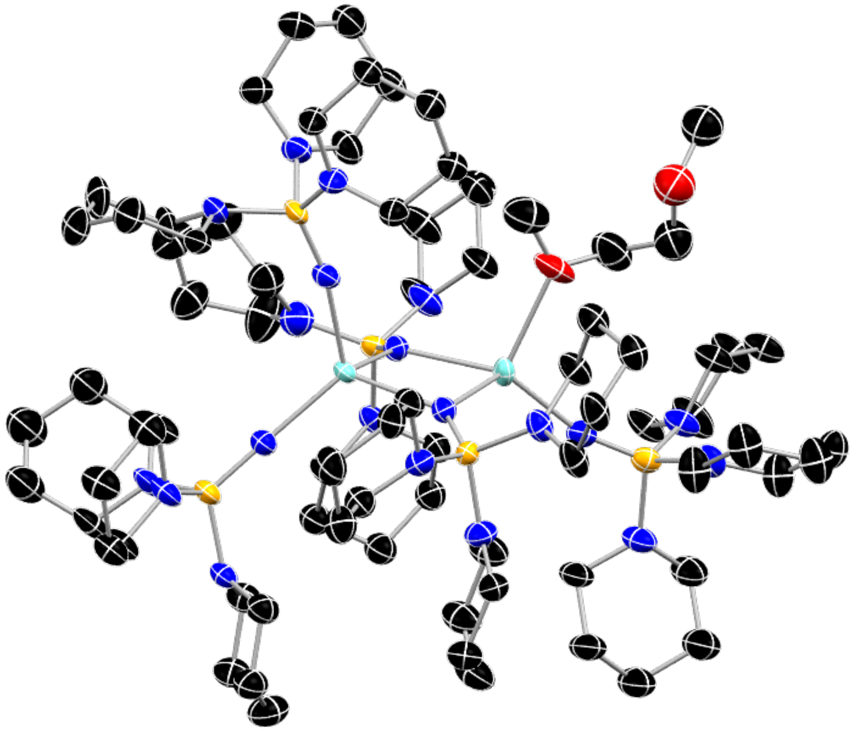

Crystal data and structure refinement for 4-$[Yb_2]^{5+}$.

Identification code	4-$[Yb_2]^{5+}$
Empirical formula	$C_{78.4}H_{158.5}I_{0.15}N_{20}O_{1.7}P_5Yb_2$
Formula weight	1928.71
Temperature/K	100(2)
Crystal system	orthorhombic
Space group	$P2_12_12_1$
a/Å	14.0554(6)
b/Å	14.0672(6)
c/Å	46.5937(19)
α/°	90
β/°	90
γ/°	90
Volume/Å^3	9212.5(7)
Z	4
ρ_{calc}g/cm^3	1.391
μ/mm^{-1}	2.208
F(000)	4022.0
Crystal size/mm^3	0.193 × 0.156 × 0.098
Radiation	MoKα (λ = 0.71073)
2Θ range for data collection/°	3.906 to 51.362
Index ranges	$-17 \leq h \leq 17$, $-17 \leq k \leq 17$, $-56 \leq l \leq 56$
Reflections collected	148747
Independent reflections	17447 [R_{int} = 0.1278, R_{sigma} = 0.0635]
Data/restraints/parameters	17447/2398/962
Goodness-of-fit on F^2	1.128
Final R indexes [I>=2σ (I)]	R_1 = 0.0734, wR_2 = 0.1489
Final R indexes [all data]	R_1 = 0.0855, wR_2 = 0.1581
Largest diff. peak/hole / e Å^{-3}	1.22/-1.28
Flack parameter	-0.078(10)

5-[Yb]$^{3+}$

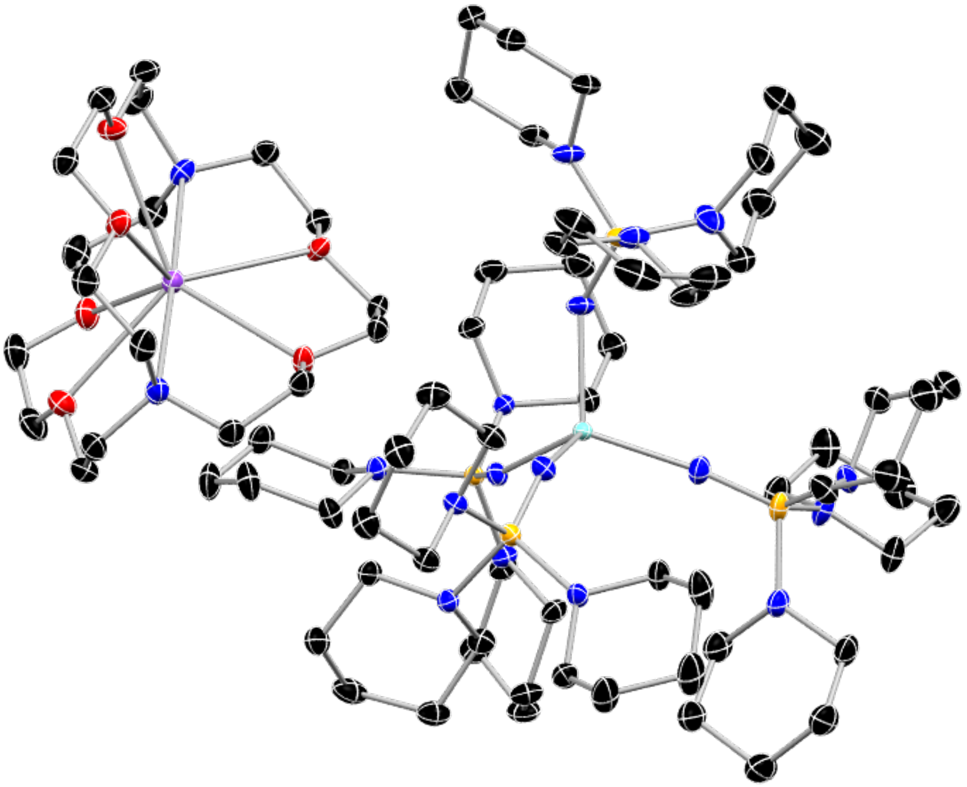

Crystal data and structure refinement for 5-[Yb]$^{3+}$.

Identification code	5-[Yb_1]$^{3+}$
Empirical formula	$C_{4.8}H_{9.71}K_{0.06}N_{1.03}O_{0.51}P_{0.23}Yb_{0.06}$
Formula weight	109.28
Temperature/K	99.99
Crystal system	triclinic
Space group	P-1
a/Å	13.9780(17)
b/Å	15.9280(16)
c/Å	23.929(3)
α/°	95.044(7)
β/°	105.496(5)
γ/°	101.582(6)
Volume/Å^3	4972.4(10)
Z	35
ρ_{calc}g/cm^3	1.277
μ/mm^{-1}	1.106
F(000)	2042.0
Crystal size/mm^3	0.411 × 0.272 × 0.222
Radiation	MoKα (λ = 0.71073)
2Θ range for data collection/°	4.36 to 56.548
Index ranges	$-18 \leq h \leq 18$, $-21 \leq k \leq 20$, $-31 \leq l \leq 30$
Reflections collected	105367
Independent reflections	24579 [R_{int} = 0.0501, R_{sigma} = 0.0405]
Data/restraints/parameters	24579/16/1079
Goodness-of-fit on F^2	1.036
Final R indexes [I>=2σ (I)]	R_1 = 0.0259, wR_2 = 0.0620
Final R indexes [all data]	R_1 = 0.0295, wR_2 = 0.0640
Largest diff. peak/hole / e Å^{-3}	0.96/-0.67

Example Calculation Input Files

TDDFT Example Input:

```
! CAM-B3LYP zora zora-DEF2-SVP AUTOAUX RIJCOSX veryslowconv notrah

%tddft
nroots 100
maxdim 20
end

%scf
maxiter 1500
DIISMaxEq 40
directresetfreq 1
DampFac 0.98
DampErr 0.05
end

%basis
newgto Yb "sarc2-zora-QZVP" end
newauxgto Yb "autoaux" end
end

*xyz {CHARGE} {MULTIPLICITY}
{Geometry Coordinates}
*
```

CASSCF Example Input (Complex 5-$[Yb]^{3+}$):

```
! DKH DKH-DEF2-SVP AUTOAUX moread

%rel
picturechange 2
end

%casscf
nel 13
norb 7
mult 2
nroots 7
trafostep rimo
printwf true
nevpt2 true
nevpt
D4Tpre 1e-13
end
rel
dosoc true
domagnetization true
dosusceptibility true
SUSStatFieldMIN 0.0
SUSStatFieldMAX 10000.0
SUSStatFieldNPoints 2
gtensor true
printlevel 3
end
```

```
end

%basis
newgto Yb "sarc2-DKH-QZVP" end
newauxgto Yb "autoaux" end
end

*xyz {CHARGE} {MULTIPLICITY}
{Geometry Coordinates}
*
```

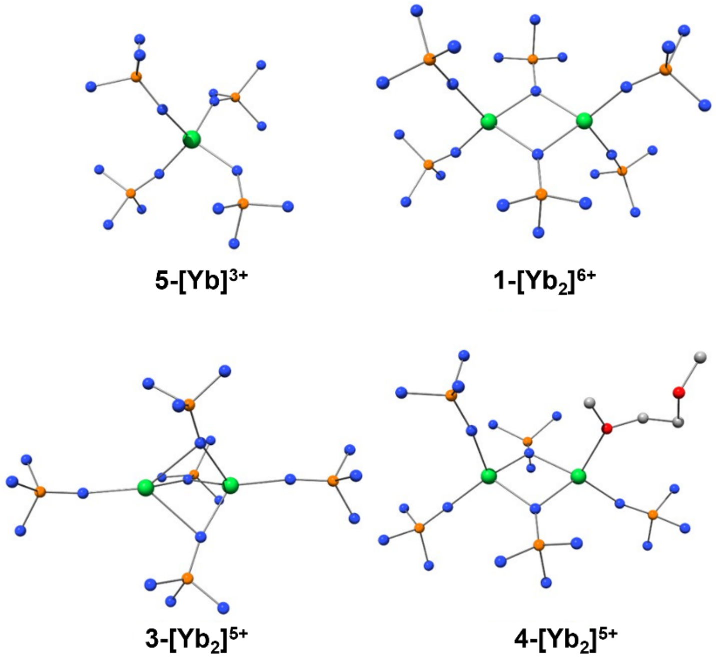

Figure S39: Model geometries used for the CASSCF calculations. Hydrogen atoms are omitted for clarity.

Model Geometries:

5-$[Yb]^{3+}$				1-$[Yb_2]^{6+}$			
Yb	0.000000000000	0.000000000000	0.000000000000	Yb	0.000000	0.000000	0.000000
P	-2.952550170900	-1.888799513300	-1.051567824200	Yb	0.000000	0.000000	3.412627
P	0.108739556400	3.025574303300	-1.780400436900	P	-2.284547	-2.208389	-1.738804
P	2.505784207400	-2.205438266600	-0.803492643200	P	1.967335	1.830156	-2.460817
P	-0.588696311700	0.622621227500	3.449659439500	P	-2.092847	2.222928	1.787310
N	-3.522988956700	-1.835774416400	-2.681048602500	P	2.284547	2.208389	5.151434
N	-4.310656953500	-1.255351968900	-0.257698346200	P	-1.967331	-1.830154	5.873445
N	-3.078997306900	-3.510002100000	-0.534319848200	P	2.092847	-2.222925	1.625318
N	-1.682788649200	-1.086765088800	-0.833754420400	N	-3.661529	-2.559083	-0.812075
N	0.766265418600	2.519715658700	-3.273404084900	N	-1.827541	-3.776923	-2.187009
N	1.092170120600	4.387826566800	-1.506858527700	N	-1.133451	-1.389066	-1.147900
N	-1.409724970700	3.590694971600	-2.258694497000	N	-2.995038	-1.494398	-3.086941
N	0.046188122000	2.088413616500	-0.582068843300	N	2.378077	0.676840	-3.643066
N	4.183043970900	-2.123906401500	-1.034243527300	N	3.489608	2.514510	-2.196072
N	2.524206443500	-3.099832362500	0.646168551000	N	1.134983	1.241328	-1.315772
N	1.884936419200	-3.329805158900	-1.890400778100	N	1.220640	3.122876	-3.239515
N	1.818050716200	-0.845042204100	-0.866353584700	N	-1.333037	3.690125	1.707545
N	0.495332852300	0.562821621800	4.778458348400	N	-3.229154	2.311357	0.565274
N	-1.025341104200	2.268957209000	3.514039562700	N	-1.088291	1.035987	1.704174
N	-2.028441716600	-0.083860166800	3.986484506100	N	-3.072136	2.315774	3.140006
N	0.000000000000	0.000000000000	2.186174024600	N	3.661529	2.559086	4.224702

H	1.370222758600	0.147231878800	4.460192854000
H	0.668336011700	1.471700021900	5.206755094400
H	-0.388360954800	2.779539066000	2.894465043200
H	-1.964695088900	2.379777686100	3.125876996700
H	-2.122894575000	-0.150043063800	5.001246832900
H	-2.101622929000	-1.010797043400	3.568913966100
H	1.584769648900	-3.102199961300	1.053242367500
H	3.134772593300	-2.622815790500	1.313475383100
H	4.384635526900	-1.297632047800	-1.597351997500
H	4.609600314100	-2.947637751700	-1.463485860900
H	1.823970965400	-4.294872616400	-1.566292670400
H	0.982812048700	-2.998382097200	-2.231358138900
H	-3.706739453200	-3.598319778500	0.262538337200
H	-2.160324880000	-3.894926430500	-0.316999023100
H	-2.779321448700	-1.415756888100	-3.239379823000
H	-3.762777847000	-2.752236397000	-3.064497396400
H	-4.865387894700	-0.682926812100	-0.893621425900
H	-3.982919821400	-0.668812642300	0.511573654900
H	0.138171598200	1.820097506500	-3.676027648400
H	1.653973826700	2.049232056200	-3.074594295200
H	-1.486368097100	4.512347269100	-2.685981207000
H	-2.091242485700	3.451343945300	-1.517682398400
H	0.998796031800	4.719894113300	-0.549485394100
H	1.060474616400	5.150372233700	-2.183660013900

N	1.827543	3.776925	5.599638
N	1.133450	1.389071	4.560527
N	2.995041	1.494400	6.499567
N	-2.378077	-0.676840	7.055697
N	-3.489610	-2.514506	5.608699
N	-1.134979	-1.241329	4.728401
N	-1.220638	-3.122875	6.652144
N	1.333040	-3.690123	1.705082
N	3.229155	-2.311355	2.847356
N	1.088291	-1.035986	1.708456
N	3.072137	-2.315772	0.272621
H	-4.017768	-1.730688	-0.329088
H	-3.472518	-3.292554	-0.124160
H	-0.819399	-3.823715	-2.341252
H	-2.338674	-4.185481	-2.972498
H	-2.586493	-0.602968	-3.361111
H	-4.005506	-1.548225	-3.211900
H	2.529108	1.052778	-4.582920
H	1.662171	-0.053093	-3.665168
H	3.473920	3.212696	-1.448463
H	4.208796	1.814492	-1.997583
H	1.801831	3.886806	-3.583807
H	0.362263	3.437316	-2.789703
H	-0.315692	3.621678	1.683226
H	-1.673279	4.441923	2.307081
H	-3.424743	1.405988	0.133501
H	-2.998946	3.004657	-0.147722
H	-4.034395	2.006765	3.001411
H	-2.672794	1.924162	3.996682
H	3.402046	2.780384	3.259286
H	4.317294	1.773649	4.219996
H	0.837010	3.968668	5.457879
H	2.432994	4.546507	5.315016
H	3.367634	2.142046	7.197802
H	2.348122	0.831582	6.929468
H	-1.651622	0.041484	7.091748
H	-2.557526	-1.049278	7.991890
H	-3.474698	-3.177757	4.829590
H	-4.208986	-1.807263	5.438145
H	-0.332861	-3.402457	6.237298
H	-1.800435	-3.910695	6.939513
H	0.315248	-3.652260	1.678102
H	1.713588	-4.404964	2.321828
H	2.996970	-1.784090	3.690684
H	4.198511	-2.172162	2.563597
H	3.246479	-1.404749	-0.161435
H	2.732345	-2.977157	-0.427804

3-$[Yb_2]^{5+}$

Yb	-0.000000000	-0.000000000	3.0077049474
Yb	0.0000000000	0.0000000000	0.0000000000
P	-1.6289592129	-2.7885513121	1.3413680870
P	0.1795154372	-0.0917338842	-3.7220972058
P	-0.3725577303	0.1672729007	6.6064926203
P	3.2653037406	-0.3660354273	1.9515577357
P	-1.2339869522	3.0628573773	1.4760021259
N	-2.6496113498	-2.5235065173	0.0788298329
N	-0.6846556077	-4.0482902503	0.7854200826
N	-2.4626262185	-3.5288563981	2.4706286220
N	-0.9720683133	-1.5471035782	1.8578635527
N	0.2384779027	-1.5265573810	-4.5238310250
N	-1.1717364595	0.6412671114	-4.3712051712
N	1.3264812268	0.8289435852	-4.3391997254
N	0.3160935479	-0.2182359473	-2.2408702528
N	-1.8909701733	0.7231822869	6.9030243997
N	-0.3167610849	-1.2623436989	7.4641097641
N	0.6229305483	1.0615118644	7.4750475470
N	0.0068202968	0.1709916589	5.1601151918
N	4.2314865648	-0.6899348499	0.6618028123
N	4.0882620149	0.8652270693	2.7171020076

4-$[Yb_2]^{5+}$

Yb	-0.019842	0.023652	3.387881
Yb	-0.018926	0.010543	0.009268
P	1.260351	1.311812	6.571260
P	2.700951	-1.411628	1.660202
P	-2.312047	-2.427660	-1.362516
P	1.654608	1.903696	-2.629170
P	-1.977176	2.363725	1.722981
O	-1.407473	-1.708152	4.162731
O	-2.003408	-4.009807	6.106401
N	0.111324	1.800528	7.684419
N	2.350323	2.589534	6.645876
N	1.965033	0.034247	7.411101
N	0.855998	0.982102	5.164603
N	3.310888	-2.138432	0.280715
N	2.785770	-2.674447	2.761750
N	3.940397	-0.343393	2.015611
N	1.332344	-0.787255	1.627601
N	-2.400268	-2.384639	-3.041316
N	-3.897862	-2.091389	-0.930495
N	-2.058937	-4.063451	-1.119083
N	-1.331079	-1.534566	-0.656799

N	3.4878245545	-1.5402583056	3.0047048096
N	1.8213989737	-0.1981604673	1.6091466614
N	-0.4244855456	3.6914152942	0.1852844094
N	-2.8403216413	3.2638196362	1.0652148829
N	-1.1200148803	4.1178957293	2.6575862976
N	-0.7488261276	1.7091605861	1.8879238150
H	-3.2919118761	-1.7462990338	0.2304397179
H	-3.0977747154	-3.3094688619	-0.3945966884
H	-0.1151214376	-4.4313036003	1.5423065926
H	-0.0770754022	-3.8080650955	-0.0036958614
H	-2.8373295413	-4.4709956004	2.3774302166
H	-2.3865387207	-3.1931312447	3.4276342796
H	-0.1242670815	-1.5991123580	-5.4759145241
H	0.0111675696	-2.3420792154	-3.9571771121
H	-2.0324003953	0.1387427235	-4.1414329494
H	-1.2611113161	1.6126229302	-4.0619878808
H	2.2019825181	0.7916777496	-3.8204575107
H	1.4724128436	0.8973077846	-5.3478707800
H	-2.2516776214	0.8172357003	7.8550066164
H	-2.6081420790	0.4002764823	6.2537594874
H	-0.8777463999	-1.3891510397	8.3054555753
H	0.5942488341	-1.7161764825	7.5305531402
H	0.6055882002	1.0666023023	8.4955355883
H	0.8951348781	1.9628797301	7.0827174667
H	3.7347937375	-0.9805658900	-0.1787459750
H	5.1012856808	-1.2001268786	0.8068806510
H	3.5875160135	1.2080132342	3.5414601955
H	4.3107000033	1.6478990686	2.0982434419
H	2.6977649812	-2.1420802988	3.2231135990
H	4.2107602246	-1.5205060449	3.7214120744
H	-0.6679223615	4.6143849095	-0.1776040377
H	0.5847865760	3.5517226625	0.2345359658
H	-3.4388096103	3.0652298343	1.8690633691
H	-3.1524119848	2.7105580268	0.2629070428
H	-0.8470213128	3.7804403433	3.5779359874
H	-1.5639126140	5.0339801245	2.6509758062

N	3.044980	2.781245	-2.287903
N	0.684379	3.078540	-3.326982
N	2.218143	0.920177	-3.869337
N	0.952501	1.166005	-1.524811
N	-1.179093	3.815693	1.997210
N	-3.092211	2.341902	2.973311
N	-2.805335	2.750842	0.316954
N	-1.177776	1.092803	1.643825
C	-2.061740	-2.620283	3.381923
C	-1.828997	-1.670758	5.520475
C	-3.137287	-4.301310	6.813208
C	-1.366298	-2.783245	6.440187
H	-2.939426	-1.611479	5.534365
H	-1.435793	-0.710744	5.914664
H	-0.620788	3.778128	2.855084
H	3.424992	-1.469309	-0.489516
H	2.370105	1.378842	-4.771367
H	-0.474948	1.034228	8.022331
H	-0.480560	2.544272	7.307296
H	2.313246	3.217422	7.449523
H	2.438199	3.087911	5.761440
H	2.543245	0.294652	8.214454
H	2.508994	-0.535800	6.759878
H	2.699810	-2.894539	-0.039743
H	3.582608	-3.311806	2.709399
H	1.905700	-3.178960	2.873058
H	4.873803	-0.713062	2.200430
H	3.663105	0.376171	2.684644
H	-1.461306	-2.505876	-3.431097
H	-2.757696	-1.480007	-3.362841
H	-4.588826	-2.833724	-1.065504
H	-3.913430	-1.746620	0.030004
H	-2.275439	-4.705817	-1.882354
H	-1.157197	-4.258724	-0.686516
H	3.840987	2.160080	-2.119647
H	2.899994	3.352081	-1.450950
H	1.110343	3.959018	-3.618752
H	-0.190267	3.209818	-2.820493
H	1.582216	0.128283	-3.979236
H	-0.570505	4.031209	1.203308
H	-3.716665	3.142119	3.085078
H	-3.589742	1.454085	3.039553
H	-3.492913	3.501707	0.307838
H	-3.043288	1.960204	-0.281712
H	-1.787645	-2.437029	2.323727
H	-3.163639	-2.502485	3.471171
H	-1.811100	-3.662168	3.652919
H	-3.522576	-5.275794	6.464928
H	-2.953080	-4.382301	7.910754
H	-1.574794	-2.484101	7.490095
H	-0.273768	-2.926318	6.341384
H	-3.956382	-3.554413	6.674257

References

(1) Izod, K.; Liddle, S. T.; Clegg, W. A Convenient Route to Lanthanide Triiodide THF Solvates. Crystal Structures of $LnI_3(THF)_4$ [Ln = Pr] and $LnI_3(THF)_{3.5}$ [Ln = Nd, Gd, Y]. *Inorg. Chem.* **2004**, *43* (1), 214-218.

(2) Rice, N. T.; Su, J.; Gompa, T. P.; Russo, D. R.; Telser, J.; Palatinus, L.; Bacsa, J.; Yang, P.; Batista, E. R.; La Pierre, H. S. Homoleptic Imidophosphorane Stabilization of Tetravalent Cerium. *Inorg. Chem.* **2019**, *58* (8), 5289-5304.

(3) Stoll, S.; Schweiger, A. EasySpin, a comprehensive software package for spectral simulation and analysis in EPR. *Journal of Magnetic Resonance* **2006**, *178* (1), 42-55.

(4) Dolomanov, O. V.; Bourhis, L. J.; Gildea, R. J.; Howard, J. A. K.; Puschmann, H. OLEX2: a complete structure solution, refinement and analysis program. *J. Appl. Crystallogr.* **2009**, *42* (2), 339-341.

(5) Sheldrick, G. SHELXT - Integrated space-group and crystal-structure determination. *Acta Crystallogr. A* **2015**, *71* (1), 3-8.

(6) Sheldrick, G. Crystal structure refinement with SHELXL. *Acta Crystallogr. C* **2015**, *71* (1), 3-8.

(7) Macrae, C. F.; Sovago, I.; Cottrell, S. J.; Galek, P. T. A.; McCabe, P.; Pidcock, E.; Platings, M.; Shields, G. P.; Stevens, J. S.; Towler, M.; et al. Mercury 4.0: from visualization to analysis, design and prediction. *J. Appl. Crystallogr.* **2020**, *53* (1), 226-235.

(8) Otte, K. S.; Niklas, J. E.; Studvick, C. M.; Boggiano, A. C.; Bacsa, J.; Popov, I. A.; La Pierre, H. S. Divergent Stabilities of Tetravalent Cerium, Uranium, and Neptunium Imidophosphorane Complexes**. *Angew. Chem. Int. Ed.* **2023**, *62* (34), e202306580.

(9) Elgrishi, N.; Rountree, K. J.; McCarthy, B. D.; Rountree, E. S.; Eisenhart, T. T.; Dempsey, J. L. A Practical Beginner’s Guide to Cyclic Voltammetry. *Journal of Chemical Education* **2018**, *95* (2), 197-206.

(10) Bain, G. A.; Berry, J. F. Diamagnetic Corrections and Pascal's Constants. *Journal of Chemical Education* **2008**, *85* (4), 532.

(11) Coak, M. J.; Liu, C.; Jarvis, D. M.; Park, S.; Cliffe, M. J.; Goddard, P. A. SquidLab—A user-friendly program for background subtraction and fitting of magnetization data. *Review of Scientific Instruments* **2020**, *91* (2), 023901.

(12) Ravel, B.; Newville, M. ATHENA, ARTEMIS, HEPHAESTUS: data analysis for X-ray absorption spectroscopy using IFEFFIT. *J. Synchrotron Rad.* **2005**, *12* (4), 537-541.

(13) Aravena, D.; Neese, F.; Pantazis, D. A. Improved Segmented All-Electron Relativistically Contracted Basis Sets for the Lanthanides. *J. Chem. Theory Comput.* **2016**, *12* (3), 1148-1156.

(14) Weigend, F.; Ahlrichs, R. Balanced basis sets of split valence, triple zeta valence and quadruple zeta valence quality for H to Rn: Design and assessment of accuracy. *Phys. Chem. Chem. Phys.* **2005**, *7* (18), 3297-3305.

(15) Yanai, T.; Tew, D. P.; Handy, N. C. A new hybrid exchange–correlation functional using the Coulomb-attenuating method (CAM-B3LYP). *Chemical Physics Letters* **2004**, *393* (1), 51-57.

(16) van Lenthe, E.; Baerends, E. J.; Snijders, J. G. Relativistic total energy using regular approximations. *J. Chem. Phys.* **1994**, *101* (11), 9783-9792.
(17) Perdew, J. P. Density-functional approximation for the correlation energy of the inhomogeneous electron gas. *Phys. Rev. B* **1986**, *33* (12), 8822-8824.
(18) Becke, A. D. Density-functional exchange-energy approximation with correct asymptotic behavior. *Phys. Rev. A* **1988**, *38* (6), 3098-3100.
(19) Heß, B. A.; Marian, C. M.; Wahlgren, U.; Gropen, O. A mean-field spin-orbit method applicable to correlated wavefunctions. *Chemical Physics Letters* **1996**, *251* (5), 365-371.
(20) Angeli, C.; Cimiraglia, R.; Evangelisti, S.; Leininger, T.; Malrieu, J. P. Introduction of n-electron valence states for multireference perturbation theory. *J. Chem. Phys.* **2001**, *114* (23), 10252-10264.
(21) Neese, F.; Wennmohs, F.; Becker, U.; Riplinger, C. The ORCA quantum chemistry program package. *J. Chem. Phys.* **2020**, *152* (22), 224108.
(22) Neese, F. Software update: the ORCA program system, version 4.0. *WIREs Computational Molecular Science* **2018**, *8* (1), e1327.
(23) Cotton, F. A. Discovering and understanding multiple metal-to-metal bonds. *Acc. Chem. Res.* **1978**, *11* (6), 225-232.
(24) Cotton, F. A.; Murillo, C. A.; Walton, R. A. *Multiple Bonds between Metal Atoms*; Springer, 2005.
(25) Pauling, L. *The Nature of the Chemical Bond*; Cornell University Press, 1960.
(26) Pyykkö, P.; Atsumi, M. Molecular Single-Bond Covalent Radii for Elements 1–118. *Chemistry – A European Journal* **2009**, *15* (1), 186-197.
(27) Cordero, B.; Gómez, V.; Platero-Prats, A. E.; Revés, M.; Echeverría, J.; Cremades, E.; Barragán, F.; Alvarez, S. Covalent radii revisited. *Dalton Trans.* **2008**, (21), 2832-2838.
(28) Lang, P. F.; Smith, B. C. Electronegativity effects and single covalent bond lengths of molecules in the gas phase. *Dalton Trans.* **2014**, *43* (21), 8016-8025.
(29) Pyykkö, P. Additive Covalent Radii for Single-, Double-, and Triple-Bonded Molecules and Tetrahedrally Bonded Crystals: A Summary. *J. Phys. Chem. A* **2015**, *119* (11), 2326-2337.
(30) Shannon, R. Revised Effective Ionic Radii and Systematic Studies of Interatomic Distances in Halides and Chalcogenides *Acta Crystallogr., Sect. A: Cryst.. Phys., Diffr., Theor. Gen. Crystallogr.* **1976**, *32*, 751-767.
(31) Wang, Y.; Del Rosal, I.; Qin, G.; Zhao, L.; Maron, L.; Shi, X.; Cheng, J. Scandium and lanthanum hydride complexes stabilized by super-bulky penta-arylcyclopentadienyl ligands. *Chem. Commun.* **2021**, *57* (63), 7766-7769.
(32) Pook, N.-P.; Adam, A. Synthesis, Crystal Structure, and Vibrational Spectra of Five Novel Peroxidocerates(IV) and the Occurrence of a New Complex Unit in $K_8[Ce_2(O_2)_3(NTA)_2]_2 \cdot 20H_2O$. *Z. Anorg. Allg. Chem.* **2014**, *640* (14), 2931-2938.
(33) Song, J.-I.; Gambarotta, S. The First Dinuclear Low-Valent Samarium Complex with a Short Sm–Sm Contact. *Angewandte Chemie International Edition in English* **1995**, *34* (19), 2141-2143.
(34) Lam, A. W.-H.; Wong, W.-T.; Wen, G.; Zhang, X.-X.; Gao, S. Synthesis, crystal structure and magnetic properties of μ_4-oxo-centered tetranuclear lanthanide clusters. *New Journal of Chemistry* **2001**, *25* (4), 531-533.

(35) Stewart, T.; Nishiura, M.; Konno, Y.; Hou, Z.; McIntyre, G. J.; Bau, R. The space between: Neutron diffraction studies reveal multiple hydrogen atom coordination numbers in an anionic dysprosium hydride cluster. *Inorg. Chim. Acta* **2010**, *363* (3), 562-566.
(36) Raeder, J.; Reiners, M.; Baumgarten, R.; Münster, K.; Baabe, D.; Freytag, M.; Jones, P. G.; Walter, M. D. Synthesis and molecular structure of pentadienyl complexes of the rare-earth metals. *Dalton Trans.* **2018**, *47* (41), 14468-14482.
(37) Fegler, W.; Venugopal, A.; Spaniol, T. P.; Maron, L.; Okuda, J. Reversible Dihydrogen Activation in Cationic Rare-Earth-Metal Polyhydride Complexes. *Angew. Chem. Int. Ed.* **2013**, *52* (31), 7976-7980.
(38) Gould, C. A.; McClain, K. R.; Reta, D.; Kragskow, J. G. C.; Marchiori, D. A.; Lachman, E.; Choi, E.-S.; Analytis, J. G.; Britt, R. D.; Chilton, N. F.; et al. Ultrahard magnetism from mixed-valence dilanthanide complexes with metal-metal bonding. *Science* **2022**, *375* (6577), 198-202.
